# Characterization of Angiotensin-Converting Enzyme 2 Ectodomain Shedding from Mouse Proximal Tubular Cells

**DOI:** 10.1371/journal.pone.0085958

**Published:** 2014-01-15

**Authors:** Fengxia Xiao, Joseph Zimpelmann, Samih Agaybi, Susan B. Gurley, Lawrence Puente, Kevin D. Burns

**Affiliations:** 1 Division of Nephrology, Department of Medicine, Kidney Research Centre, Ottawa Hospital Research Institute, University of Ottawa, Ottawa, Ontario, Canada; 2 Division of Nephrology, Department of Medicine, Duke University Medical Centre, Durham, North Carolina, United States of America; 3 Sprott Center for Stem Cell Research, Ottawa Hospital Research Institute, Ottawa, Ontario, Canada; Max-Delbrück Center for Molecular Medicine (MDC), Germany

## Abstract

Angiotensin-converting enzyme 2 (ACE2) is highly expressed in the kidney proximal tubule, where it cleaves angiotensin (Ang) II to Ang-(1-7). Urinary ACE2 levels increase in diabetes, suggesting that ACE2 may be shed from tubular cells. The aim of this study was to determine if ACE2 is shed from proximal tubular cells, to characterize ACE2 fragments, and to study pathways for shedding. Studies involved primary cultures of mouse proximal tubular cells, with ACE2 activity measured using a synthetic substrate, and analysis of ACE2 fragments by immunoblots and mass spectrometry. The culture media from mouse proximal tubular cells demonstrated a time-dependent increase in ACE2 activity, suggesting constitutive ACE2 shedding. ACE2 was detected in media as two bands at ∼90 kDa and ∼70 kDa on immunoblots. By contrast, full-length ACE2 appeared at ∼100 kDa in cell lysates or mouse kidney cortex. Mass spectrometry of the two deglycosylated fragments identified peptides matching mouse ACE2 at positions 18-706 and 18-577, respectively. The C-terminus of the 18-706 peptide fragment contained a non-tryptic site, suggesting that Met^706^ is a candidate ACE2 cleavage site. Incubation of cells in high D-glucose (25 mM) (and to a lesser extent Ang II) for 48–72 h increased ACE2 activity in the media (p<0.001), an effect blocked by inhibition of a disintegrin and metalloproteinase (ADAM)17. High D-glucose increased ADAM17 activity in cell lysates (p<0.05). These data indicate that two glycosylated ACE2 fragments are constitutively shed from mouse proximal tubular cells. ACE2 shedding is stimulated by high D-glucose, at least partly via an ADAM17-mediated pathway. The results suggest that proximal tubular shedding of ACE2 may increase in diabetes, which could enhance degradation of Ang II in the tubular lumen, and increase levels of Ang-(1-7).

## Introduction

Angiotensin-converting enzyme 2 (ACE2) is a component of the renin-angiotensin system that contains a single HEMGH zinc-dependent catalytic site, degrading the vasoconstrictor angiotensin (Ang) II to the vasodilator Ang-(1-7) [1], [2]. Although ACE2 is found in many tissues, it is highly expressed in the kidney, particularly within cells of the proximal tubule (PT) [3], [4]. Experimental studies suggest that ACE2 protects against renal disease progression. Thus, ACE2 gene knockout (KO) mice develop accelerated Ang II-mediated glomerulosclerosis [5] and are more susceptible to kidney injury in the type 1 diabetes Akita model [6]. Moreover, in Akita diabetic mice, administration of exogenous human recombinant ACE2 attenuates blood pressure and glomerular injury [7]. We recently reported that podocyte-specific overexpression of human ACE2 attenuates streptozotocin (STZ)-induced diabetic nephropathy in mice [8]. In kidney biopsies from patients with type 2 diabetes and kidney disease, glomerular and tubular expression of ACE2 is decreased, which may result in increased Ang II levels and subsequent enhanced renal injury [9]. In contrast, mice with diabetic nephropathy exhibit diminished glomerular ACE2 expression, but increased tubular ACE2, suggesting a compensatory mechanism to counteract the effects of increased Ang II [3], [10].

ACE2 is a type I integral membrane protein that shares 42% homology with angiotensin-converting enzyme (ACE) in its *N*-terminal extracellular catalytic domain [2]. Unlike ACE, however, ACE2 is not blocked by ACE inhibitors [2]. ACE2 may be acted upon by metalloproteases, resulting in cleavage and extracellular shedding at its C-terminus from the cell surface [11]-[13]. Indeed, ectodomain shedding of soluble forms of ACE2 has been reported to occur in human embryonic kidney (HEK) cells and airway epithelial cells, mediated by the enzyme tumor necrosis factor (TNF)-α convertase (TACE), also known as ADAM17 (a member of the “A Disintegrin And Metalloproteinase” family) [11]–[13].

Soluble ACE2 was first reported in human urine by immunoblot in 2005 [14]. Urinary ACE2 levels increase in db/db diabetic mice and mice with STZ-diabetes, an effect that does not appear to be due to passage of the enzyme across the glomerular filtration barrier [15]. Furthermore, urinary ACE2 levels increase in chronic kidney disease (CKD) patients with diabetes [16], [17], and are closely associated with type 2 diabetes mellitus in humans [18]. In these studies, urinary ACE2 fragments have been detected of smaller molecular mass compared to full-length ACE2 [15]–[18]. Recently Chodavarapu et al. demonstrated increased renal expression of ADAM17 in diabetic db/db mice, in association with increased urinary ACE2 excretion [19]. Treatment with the insulin sensitizer rosiglitazone decreased renal ADAM17 expression and urinary ACE2 levels [19]. Accordingly, these data suggest that ACE2 may be shed from tubular cells into the urinary space, a process that may be enhanced in diabetes via activation of ADAM17. In this regard, PT cells represent a potential source for urinary ACE2. However, the effects of high extracellular glucose or Ang II on PT ACE2 shedding, and the role of ADAM17 in these cells have not been explored. Furthermore, ACE2 fragments shed from PT cells have not been characterized with regards to possible cleavage sites within ACE2.

The aim of this study was therefore to first determine if ACE2 is shed from primary cultures of mouse PT cells, and to characterize shed ACE2 fragments by immunoblot analysis and mass spectrometry. Importantly, the effect of high D-glucose or Ang II on ACE2 ectodomain shedding was examined, as was the role of ADAM17. The results reveal that two glycosylated ACE2 fragments of ∼90 and ∼70 kDa are constitutively shed from mouse PT cells, and that high glucose and, to a lesser extent Ang II, stimulate shedding via an ADAM17-mediated pathway.

## Materials and Methods

### Ethics statement

This study involved primary cultures of mouse proximal tubular cells as described below, and all mice were housed according to the Canadian Council on Animal Care (CCAC) guidelines. The experimental protocols were approved by the Animal Care Committee at the University of Ottawa.

### Primary culture of mouse PT cells

Male C57BL6 mice were obtained from Charles River Laboratories (Saint-Constant, Quebec, Canada), and ACE2 gene KO mice (C57BL6 background) were obtained from Duke University Medical Center (Durham, NC, USA). For each experiment, primary cultures of PT cells were prepared from 2 male C57BL6 or ACE2 KO mice (4 kidneys, age of mice 12–16 weeks) by collagenase digestion of renal cortices followed by Percoll gradient centrifugation, essentially as described [20]. Briefly, kidneys were harvested aseptically from anesthetized mice, and renal cortices were dissected, gently minced, and suspended in a perfusion solution consisting of (in mM) 105 NaCl, 24 NaHCO_3_, 5.0 KCl, 2.0 Na_2_HPO_4_, 1.0 MgSO_4_, 1.5 CaCl_2_, 4.0 Na lactate, 5.0 D-glucose, 1.0 L-alanine, 10 *N*-2-hydroxyethylpiperazine-*N*-2-ethanesulfonic acid (HEPES), 0.2% bovine serum albumin (BSA), 0.1% collagenase (type V; Sigma, St. Louis, MO, USA), and 0.05% soybean trypsin inhibitor (Sigma), pH 7.2. The suspension was gassed with 95% O_2_-5% CO_2_ for 30 min at 37°C. After collagenase digestion, the cortical suspension was strained through a 250 µm brass sieve and centrifuged at 1500 g for 1 min. The pellet was washed with phosphate-buffered saline (PBS), and resuspended in a 40% Percoll solution (Sigma) (pH 7.2) containing (in mM) 120 NaCl, 4.8 KCl, 25 NaHCO_3_, 1.0 MgCl_2_, 1.0 NaH_2_PO_4_, 5.0 D-glucose, 10 HEPES, 1.0 L-alanine, 1.4 CaCl_2_, 60 U/ml penicillin, and 60 µg/ml streptomycin. The solution was centrifuged at 18500 g for 30 min at 4°C, and the digested tissue separated into four distinct bands. The fourth band (F4), enriched in PT segments, was aspirated, suspended in culture medium and centrifuged to remove the Percoll solution. For 2 mice, the final PT pellet was suspended in culture medium and seeded onto 32 35-mm culture dishes.

Cells were initially grown in a defined medium of DMEM-F12 (1∶1), supplemented with insulin (5 µg/ml), transferrin (5 µg/ml), selenium (5 ng/ml), hydrocortisone (50 nM), 3,3′,5-triiodo-*L*-thyronine (2.5 nM), 100 U/ml penicillin, 100 mg/ml streptomycin, and 10% fetal bovine serum (FBS). After 24 h, cells were attached to the culture dishes, and switched to a defined serum-free medium, with glucose concentration 7.8 mM. After 5 days, cells reached ∼70% confluence and had a typical epithelial cell morphology, with cobblestone appearance. Cells were maintained at 37°C in a humidified incubator with 5% CO_2_/room air, and the medium was changed every 2-3 days up to the time of experimentation, which was typically after 7 days in culture. In experiments that assessed time-dependent accumulation of ACE2 activity in the conditioned media, media was changed on day 2 after plating, with no subsequent change in media for up to 6 days in culture. Cells remained viable and retained their cobblestone morphology with this maneuver.

In some experiments, subconfluent PT cells were incubated for 24–72 hrs with varying concentrations of Ang II (10^−10^–10^−7^ M, added to the culture dish every 24 hrs to offset the effect of possible degradation) (Bachem Bioscience Inc., King of Prussia, PA, USA), the AT_1_ receptor antagonist losartan (10^−5^ M, Merck Research Laboratories, Rathway, NJ, USA), high levels of D-glucose (25 mM), or L-glucose (25 mM). Some cells were also treated with the inhibitors of ADAM17, TNF-α Protease Inhibitor-1 (TAPI-1; 10^−7^–10^−5^ M) (Calbiochem, San Diego, CA, USA), TNF-α Protease Inhibitor-2 (TAPI-2; 10^−6^–5×10^−5^ M) (Calbiochem), or with the matrix metalloproteinase (MMP) inhibitor *N*-[(2R)-2-(hydroxyamidocarbonylmethyl)-4-methylpentanoyl]-*L*-tryptophan methylamide, dissolved in DMSO (GM6001 (5×10^−5^ M), Millipore Corp., Billerica, MA, USA). All vehicle controls with use of GM6001 consisted of cells exposed to an equivalent amount of DMSO, which did not affect ACE2 activity in the media compared to non-DMSO treated cells.

### Cell transfection

Primary cultures of PT cells derived from ACE2 KO mice were transiently transfected with a human ACE2 expression vector (HA-hACE2), in which the cDNA encoding the full-length human ACE2 gene was cloned into the XhoI restriction site of the expression vector pcDNA3 (Invitrogen, Carlsbad, CA, USA) [8]. As a negative control, some cells were transfected with an empty pcDNA3 vector. One day prior to transfection, subconfluent PT cells (50–70%) were placed in defined medium without antibiotics. Cells were then transiently transfected with 3.75 µg of HA-hACE2 plasmid or empty pcDNA3 vector on 35-mm culture dishes, using Lipofectamine™ LTX reagent, as per the supplier protocol (Invitrogen), and after 48 hrs, ACE2 activity or protein expression in cell lysates and culture medium was assayed. The time point of 48 hrs for measurement of ACE2 activity was chosen based on preliminary experiments, in which transient transfection of PT cells derived from wild-type mice with HA-hACE2 resulted in significant increases in ACE2 activity in the media after 48 hrs (p<0.001 vs untransfected controls or cells transfected with empty pcDNA3 vector; n = 3), but no detectable increase in activity at 72, 96 or 120 hrs.

### ACE2 enzymatic activity assay

ACE2 activity in cell culture media and membrane fractions was measured using a commercially available synthetic fluorogenic substrate for ACE2 (Mca-Ala-Pro-Lys(Dnp)-OH) (AnaSpec, San Jose, CA, USA), essentially as described [15], [17], [21]. Cell media was collected, followed by addition of a protease inhibitor cocktail (1% vol/vol) (Sigma, P8340). For all experiments, media was first centrifuged at 9300 g for 5 min at 4°C to remove dead cells and cellular debris. The cell membrane fraction from PT cells was isolated as previously described [8]. Cell media (15 µL) or membrane fractions (1-2 µg) were then added to the wells of a 96-well plate (total volume 100 µL/well) in a solution containing 37.5 mM MES, 225 mM NaCl, 7.5 µM ZnCl_2_, 0.75 mM *N*-ethylmaleimide (NEM), 0.75 mM phenylmethylsulfonyl fluoride (PMSF), 11.25 µM ACE2 substrate, with or without 1 µM of the ACE2 inhibitor MLN-4760 (GL1001, provided by Ore Pharmaceuticals, Cambridge, MA, USA). In this assay, MLN-4760 consistently caused at least 95% inhibition of ACE2 activity in conditioned media samples (see Table S1). Samples were protected from light and incubated for 16 hrs on a plate shaker at room temperature and fluorescence was measured using the FLUOstar Galaxy fluorometer (BMG Labtech, Durham, NC, USA) with excitation wavelength of 320 nM and emission wavelength of 405 nM. Preliminary experiments determined that for time points up to 16 hrs of incubation with ACE2 fluorogenic substrate, the linear range of detection for mouse recombinant ACE2 was between 1.56–50 ng/ml (R^2^ = 0.9976, p<0.001). Further experiments determined that using this assay, ACE2 activity in media samples from primary cultures of mouse PT cells was linear for at least 24 hrs of incubation with the fluorogenic substrate (Figure S1), and therefore 16 hrs of incubation was chosen for data collection. ACE2 activity was determined by subtracting the Relative Fluorescence Unit (RFU) obtained in the presence of MLN-4760 from the reading in the absence of inhibitor. ACE2 activity in the culture medium was corrected for the cell protein amounts on the culture dishes, and is reported as RFUs per µg protein per hour (RFU/µg/hr).

### ADAM17 activity assay

Cell lysates were prepared in a buffer containing 1% Nonidet P40, 10 µM pepstatin, 10 µM leupeptin, 1 µg/ml soybean trypsin inhibitor, 100 U/ml trasylol, 0.2 mg/ml α-1-antitrypsin, 1 mM PMSF, and 25 mM Tris HCl (pH 7.4). Samples were sonicated for 1 sec, and incubated on ice for 1 hr, followed by centrifugation at 6000 g for 10 min at 4°C to remove insoluble debris. Protein from the supernatant (25 µg) was used to determine ADAM17 activity with the InnoZyme™ TACE Activity Kit (Calbiochem) [22]. A standard curve was generated for the assay, using recombinant human ADAM17 provided with the kit. The amount of ADAM17 was corrected for total protein, and reported as ng/µg protein.

### ACE2 immunoblot assays

Cell culture medium was removed from the dish, subjected to centrifugation to remove dead cells and insoluble debris as described above, and concentrated with the Nanosep 30 K Centrifugal Device (Pall Corporation, Port Washington, NY, USA). The concentrate (25 µl) was then prepared in a buffer consisting of 31.3 mM Tris-HCl (pH 6.8), 1% wt/vol SDS, 5% glycerol, and 0.025% wt/vol bromophenol blue, and boiled for 5 min. Lysates from PT cells were prepared in a buffer consisting of 62.5 mM Tris·HCl (pH 6.8), 2% wt/vol SDS, 10% glycerol, 50 mM DTT, and 0.01% wt/vol bromophenol blue, boiled for 5 min, and centrifuged at 9300 g for 5 min at 4°C to remove insoluble debris.

Twenty-five µls of concentrated media (50-fold concentrate), 30–50 µg of protein from cell lysates and 1.5–10 µg of protein from mouse kidney cortex were run on 7.5% SDS-polyacrylamide gels, and subjected to immunoblot analysis using commercially available goat anti-human ACE2 antibodies (1∶500 dilution) (AF933, R&D Systems Inc.) as we previously described [23]. Densitometric analysis of the protein bands was performed using Kodak ID image analysis software (Eastman Kodak, Rochester, NY, USA).

In some experiments, prior to immunoblot analysis for ACE2, concentrated medium and cell lysates were subjected to deglycosylation using peptide *N*-glycosidase F (PNGase F, New England Biolabs, Ipswich, MA, USA), according to the manufacturer's instructions. Samples were denatured in 0.5% SDS, 40 mM DTT at 100°C for 10 min. After cooling to room temperature, the samples were incubated in 50 mM sodium phosphate (pH 7.5), 1% NP-40 (vol/vol), and 1000 U PNGase F at 37°C for 3 hrs.

### Immunoprecipitation of ACE2 protein

Cell culture medium was concentrated 200-fold by ultrafiltration with Amicon Ultra-15 30 K Centrifugal Filter Devices (Millipore), followed by immunoprecipitation with the Pierce Classic IP kit (Pierce Biotech, Rockford, IL, USA). Briefly, 200 µL of concentrated cell medium (containing protease inhibitor cocktail (Sigma)) was incubated with 6.0 µg of goat anti-human ACE2 antibody (R&D Systems Inc.) in 400 µL of IP lysis/wash buffer at 4°C for 16 hrs. The antigen-antibody complex was captured by adding the sample to a spin column containing 20 µL of Protein A/G resin, followed by incubation for 1 hr at 4°C. After several washes, the captured sample was eluted, followed by deglycosylation with PNGase F. Concentrated culture medium from PT cells derived from ACE2 KO mice was applied as a negative control for nonspecific binding of antigen-antibody.

### Mass Spectrometry of ACE2 protein

The deglycosylated immunoprecipitated ACE2 polypeptides from conditioned cell culture medium were separated on 10% SDS-polyacrylamide gels, and subjected to silver staining using SilverQuest silver staining kit (Invitrogen). The polypeptide bands matching those identified on immunoblots were excised and destained. Proteins were in-gel digested using trypsin (Promega, Madison, WI, USA), as described [24]. The resulting peptide extracts were purified by ZipTip (Milllipore), concentrated by vacufuge (Eppendorf, Hamburg, Germany), and resuspended in 0.1% formic acid (Thermo Fisher Scientific, Waltham, MA, USA).

Peptides were analyzed by Liquid Chromatography-Tandem Mass Spectrometry (LC-MS/MS) on an UltiMate 3000 RSLC nano HPLC (Dionex, Sunnyvale, CA, USA) and an LTQ Orbitrap XL hybrid mass spectrometer with nanospray ionization source (Thermo Fisher Scientific) at the Ottawa Hospital Research Institute Proteomics Core Facility (Ottawa, Ontario, Canada). The system was controlled by Xcalibur software version 2.0.7 (Thermo Fisher Scientific). Peptides were loaded onto a C18 CapTrap column (Michrom Bioresources, Auburn, CA, USA) for 5 min at a flow rate of 15 µL/min and then eluted over a 60 min gradient of 3%–45% acetonitrile with 0.1% formic acid at a flow rate of 0.3 µL/min onto a 10-cm long column with integrated emitter tip [Picofrit PF360-75-15-N-5 (New Objective, Woburn, MA, USA) packed with Zorbax SB-C18 5 micron (Agilent, Santa Clara, CA, USA)], and nanosprayed into the mass spectrometer. MS scans were acquired in FTMS mode at a resolution setting of 60,000. MS^2^ scans were acquired in ion trap CID mode using data-dependent acquisition of the top 5 ions from each MS scan.

MASCOT software version 2.4 (Matrix Science, Boston, MA, USA) was used to infer peptide and protein identities from the mass spectra. The observed MS/MS spectra were matched against a custom database of protein sequences (mouse sequences from the 2011_07 version of uniprot_sprot.fasta.gz downloaded from ftp.uniprot.org concatenated with a Contaminants database downloaded from maxquant.org, June 9th 2011). Mass tolerance parameters were MS±7 ppm and MS/MS±0.6 Da.

### Statistics

Data are presented as mean ± S.E. Data were analyzed using SigmaStat (version 3.01A; SYSTAT). For multiple comparisons, analysis was by one-way analysis of variance followed by Bonferroni correction. For comparisons involving two groups, Student's t-test was used. A p value < 0.05 was considered significant.

## Results

### Ectodomain shedding of ACE2 in mouse PT cells

**Figure 1:**
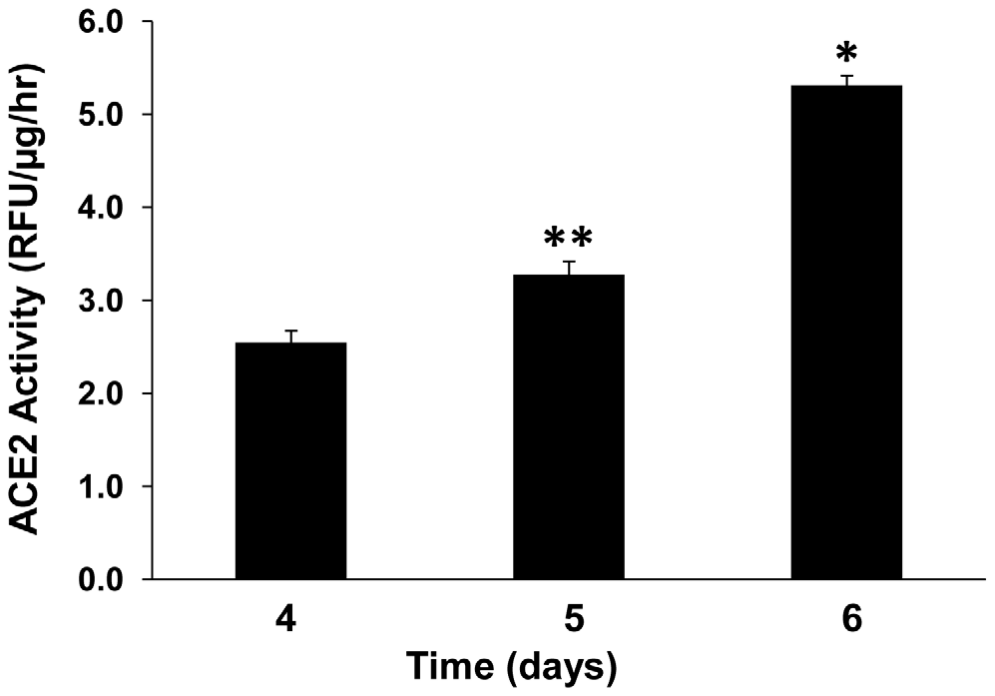
Increased ACE2 activity in media from mouse proximal tubular cells. A time-dependent increase in ACE2 activity occurred in the cell media. Results are means ± SE, corrected for total cell protein from culture dishes set up in parallel to those where media was analyzed for ACE2 activity on three successive days. *P<0.001 vs day 4 and day 5, and **P<0.01 vs day 4, n = 4.

**Figure 2:**
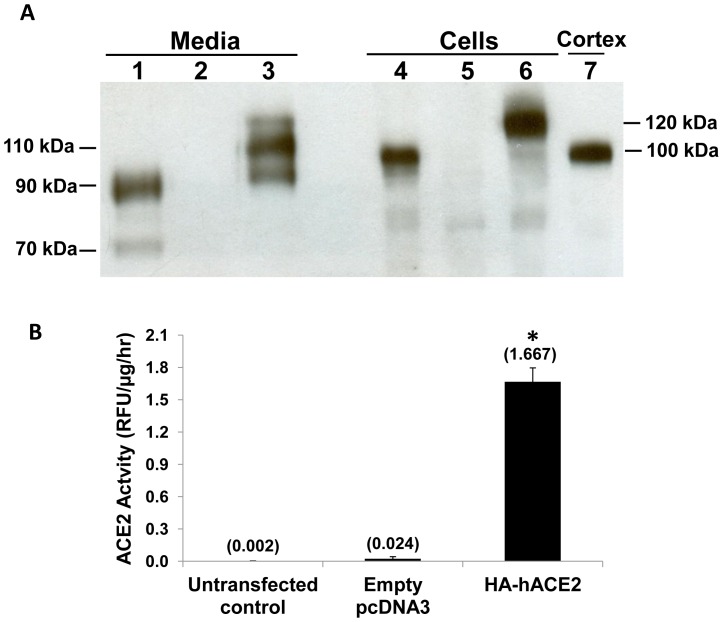
Immunoblot analysis of ACE2 protein in media and cell lysates from mouse PT cells. (A) Representative immunoblot for ACE2 protein in concentrated media (Lanes 1–3) and cell lysates (Lanes 4–6) from mouse PT cells. Lanes 1 and 4: wildtype cells, Lanes 2 and 5: ACE2 knockout (KO) cells, Lanes 3 and 6: ACE2 KO cells transfected with a human ACE2 expression vector, Lane 7: mouse kidney cortex showing a band at ∼100 kDa, used as a positive control. Lane 1 shows two bands in the media at ∼90 kDa and ∼70 kDa for mouse ACE2. Lane 3 shows two bands in the media for human ACE2 in transfected cells, at ∼110 kDa and ∼95 kDa. Lanes 4 and 6 show a single band in cell lysates at ∼100 kDa for mouse ACE2, and ∼120 kDa for human ACE2, respectively. Lanes 2 and 5 show no ACE2 bands detected on immunoblots of both media and cell lysates from untransfected ACE2 KO cells. (B) Increased ACE2 activity in the media from ACE2 KO cells transfected with a human ACE2 expression vector (HA-hACE2, 3.75 µg on 35 mm culture dishes). Untransfected cells and cells transfected with an empty pcDNA3 vector had no detectable ACE2 activity in the media. Numbers in parentheses represent mean values for ACE2 activity. *P<0.001 vs untransfected control or empty pcDNA3 vector, n = 4.

ACE2 is a type 1 integral membrane protein with a catalytically active ectodomain, which can be shed from the cell surface and retain its activity in the extracellular space [11]–[13]. In primary cultures of mouse PT cells, a time-dependent increase in ACE2 enzymatic activity was detected in the media, suggesting constitutive shedding (Figure 1). As shown in Figure 2A, in lysates from primary cultures of mouse PT cells, ACE2 was detected as a protein of ∼100 kDa by immunoblot, consistent with its size as detected in mouse kidney cortex [23]. However, in concentrated media from mouse PT cells, immunoblot analysis revealed 2 fragments for ACE2, at ∼90 kDa and ∼70 kDa. Further experiments determined if ACE2 fragments could be shed from PT cells derived from ACE2 gene KO mice, transfected with a plasmid expressing the human ACE2 cDNA. ACE2 was not detected by immunoblot in either cell lysates or media from untransfected PT cells derived from ACE2 KO mice. By contrast, transfection with the human ACE2 plasmid resulted in detection of the expected single band for human ACE2 at ∼120 kDa in cell lysates [17], while the cell media contained 2 bands at ∼110 kDa and ∼95 kDa (Figure 2A). Furthermore, while untransfected cells and cells transfected with an empty pcDNA3 plasmid demonstrated no measurable ACE2 activity in the media, cells transfected with the human ACE2 cDNA had significant levels of ACE2 activity in the media after 48 hrs (Figure 2B; P<0.001 vs untransfected control or empty pcDNA3 vector; n = 4).

### Deglycosylation and LC-MS/MS analysis of mouse ACE2 fragments

**Figure 3:**
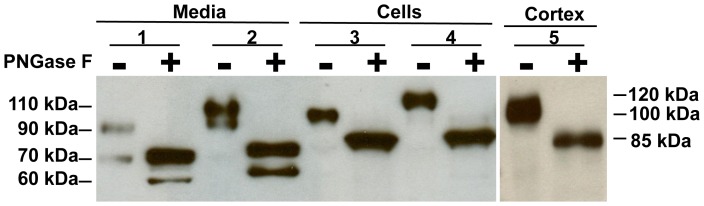
Deglycosylation of ACE2 protein in media and cell lysates from mouse PT cells. Representative immunoblot for ACE2 treated without (−) or with (+) deglycosylation with PNGase F in the media (Lanes 1–2) and cell lysates (Lanes 3–4). Lanes 1 and 3: wildtype PT cells, Lanes 2 and 4: ACE2 knockout (KO) PT cells transfected with a human ACE2 vector, Lane 5: mouse kidney cortex. Lanes 1+ and 2+ show a reduction in the sizes of ACE2 fragments in media fractions to ∼75 kDa and ∼60 kDa for mouse ACE2, and to ∼80 kDa and ∼65 kDa for human ACE2, respectively. Lanes 3+ and 4+ show a reduction in the sizes of ACE2 in cell lysates to ∼85 kDa for both mouse and human ACE2 treated with the PNGase F, respectively. Lane 5+ shows a reduction in size of ACE2 in mouse cortex from ∼100 kDa to ∼85 kDa after treatment with PNGase F.

Cell media derived from mouse PT cells was incubated with the enzyme PNGase F, to determine if shed ACE2 fragments were glycosylated. As shown in Figure 3, in PT cell lysates full-length ACE2 decreased in size from ∼100 kDa to ∼85 kDa with deglycosylation. Similarly, in mouse kidney cortex lysates, the size of ACE2 was reduced from ∼100 kDa to ∼85 kDa after treatment with PNGase F. Deglycosylation decreased the size of mouse ACE2 fragments in the cell media from ∼90 kDa and ∼70 kDa to ∼75 kDa and ∼60 kDa, respectively. In cell lysates derived from mouse ACE2 KO PT cells transfected with the human ACE2 cDNA, a reduction in ACE2 size from ∼120 kDa to ∼85 kDa was observed with deglycosylation. Human ACE2 fragments in the cell media also decreased in size with deglycosylation from ∼110 kDa and ∼95 kDa to ∼80 kDa and ∼65 kDa, respectively.

**Figure 4:**
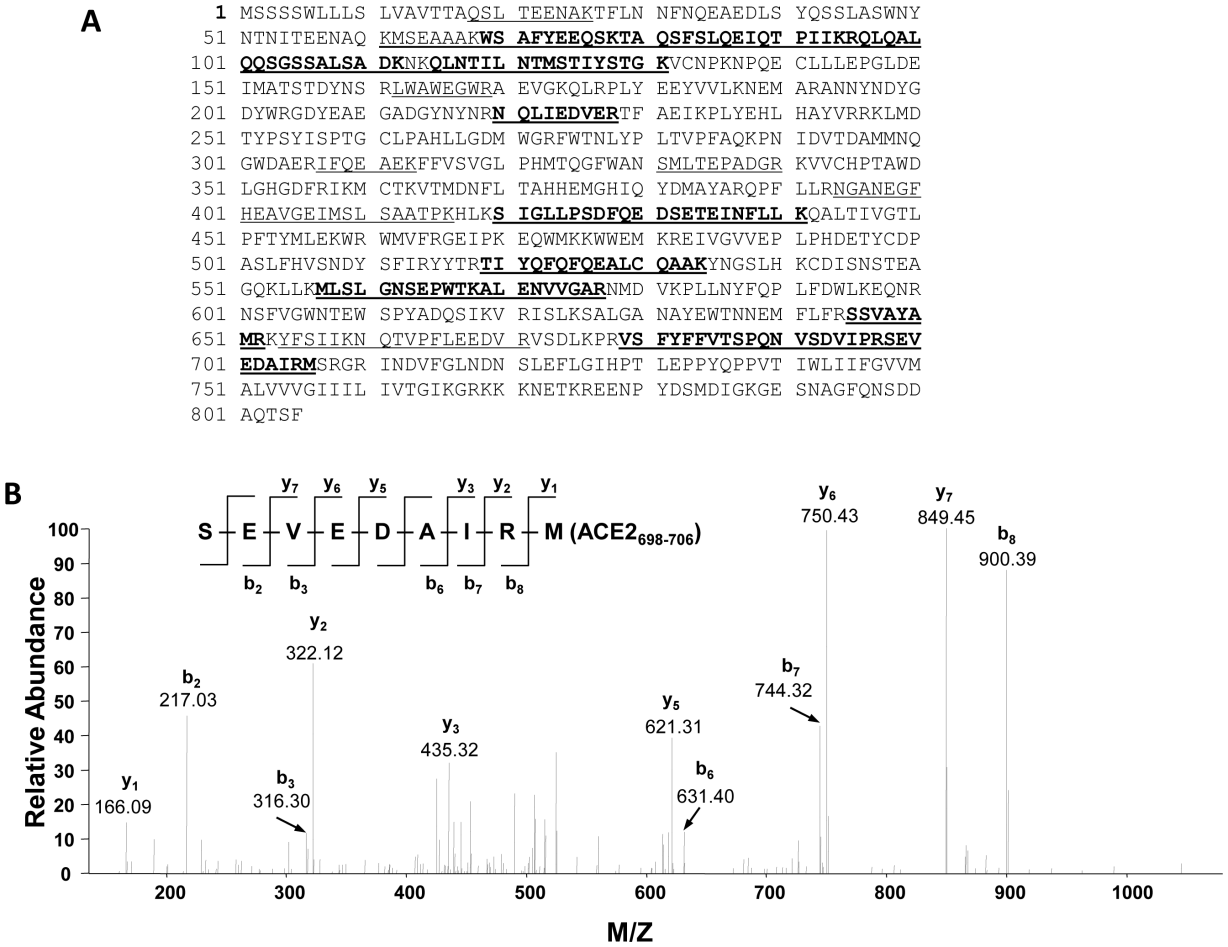
Identification of ACE2 peptides by LC-MS/MS in the 75 kDa deglycosylated band from the media of mouse PT cells. (A) Mass spectrometry identified peptides matched with the mouse ACE2 sequence (SWISS-PROT database no. Q8R0I0) at positions 18-706. The identified peptide sequences significantly matched with mouse ACE2 precursor in the database are underlined and shown in bold. The peptides matched with mouse ACE2 sequence, but not statistically significant, are underlined only (see Table S2 for detailed analyses of peptides). The overall matched sequences cover 32% of mouse ACE2 sequences. (B) Tandem mass spectrum of the *C*-terminal peptide (SEVEDAIRM 698–706). The b and y ions result from the cleavage of peptide bonds and correspond to *N*-terminal and *C*-terminal fragments of the peptide, respectively. The detected b and y ions are consistent with the peptide sequence shown on the top part of the panel. Ions score: 50, Observed ion: 533.25, Mr(expt): 1064.48, Mr(calc): 1064.48. Expect value: 0.002 (see Table S2 for details). The amino acid Met^706^ detected is from non-tryptic cleavage, suggesting a cleavage site for the ectodomain shedding of mouse ACE2 into the media.

Experiments were conducted to identify the ACE2 peptide fragments shed from primary cultures of mouse PT cells. Concentrated media samples from mouse PT cells were immunoprecipitated with an ACE2 antibody, treated with PNGase F, and separated on silver-stained SDS-PAGE gels. Trypsin digestion of the ∼75 kDa mouse ACE2 fragment was followed by LC-MS/MS analysis, which identified peptides matched in sequence with mouse ACE2 from amino acid positions 18 to 706, corresponding to a polypeptide with a predicted molecular mass of 79.7 kDa (Figure 4 and Table S2). The C-terminus Met^706^ detected in the ∼75 kDa fragment represents a site of non-tryptic cleavage, and is a candidate site for ectodomain cleavage of ACE2. LC-MS/MS analysis of the immunoprecipitated ∼75 kDa fragment from 3 additional separate experiments revealed ACE2 peptides with the C-terminus as Arg^705^, a tryptic cleavage site.

**Figure 5:**
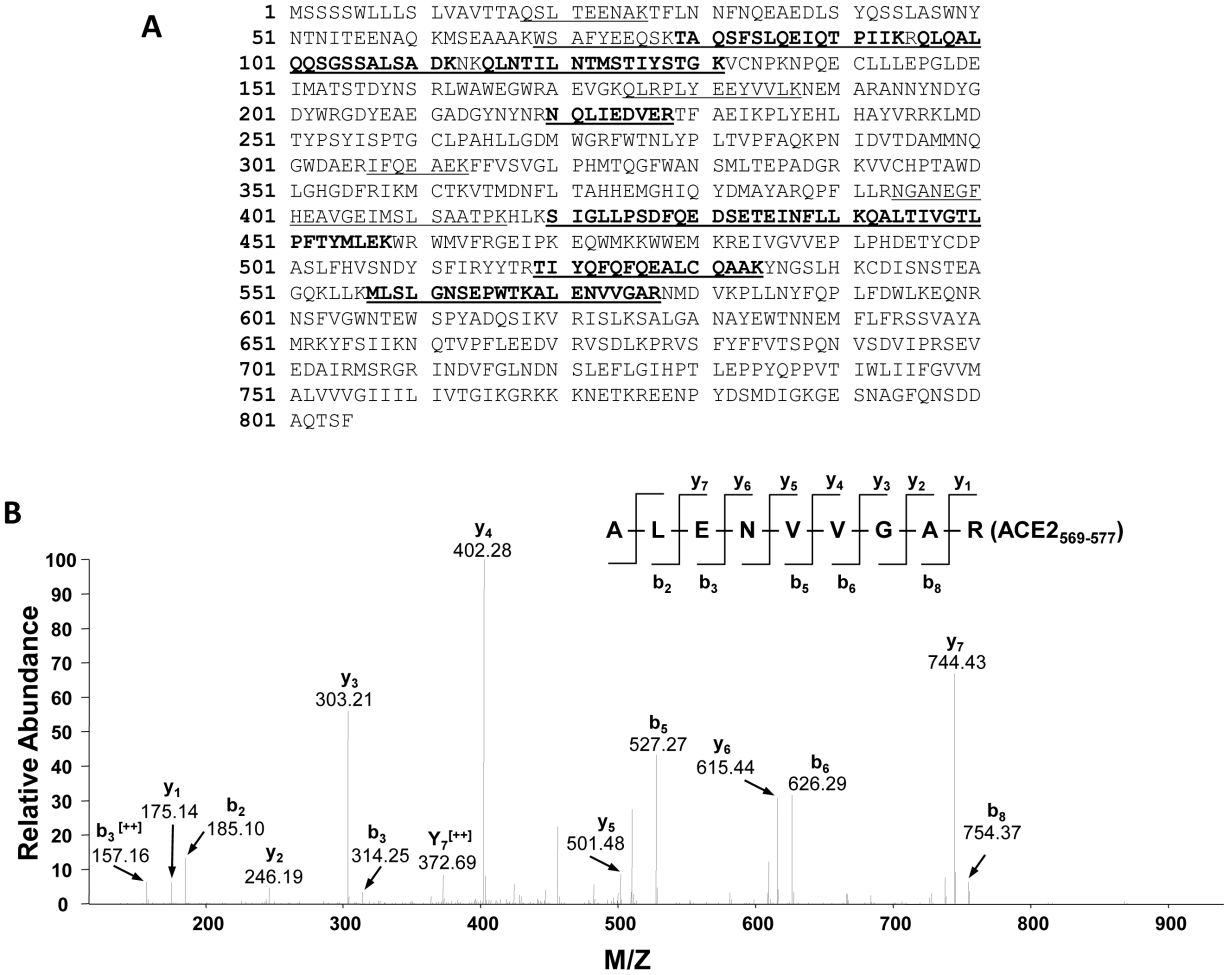
Identification of ACE2 peptides by LC-MS/MS in the 60 kDa deglycosylated band from the media of mouse PT cells. (A) Mass spectrometry identified peptides matched with the mouse ACE2 sequence (SWISS-PROT database no. Q8R0I0) at positions 18-577. The identified peptides significantly matched with mouse ACE2 sequences are underlined and shown in bold. The peptides matched with mouse ACE2 sequence, but not statistically significant, are underlined only (see Table S3 for detailed analyses of peptides). The overall sequence coverage is 24%. (B) Tandem mass spectrum of the *C*-terminal peptide (ALENVVGAR 569-577). The b and y ions result from the cleavage of peptide bonds and correspond to *N*-terminal and *C*-terminal fragments of the peptide, respectively. The detected b and y ions are consistent with the peptide sequence shown on the top part of the panel. Ions score: 59, Observed ion: 464.77, Mr(expt): 927.52, Mr(calc): 927.51. Expect value: 0.0002 (see Table S3 for details).

**Figure 6:**
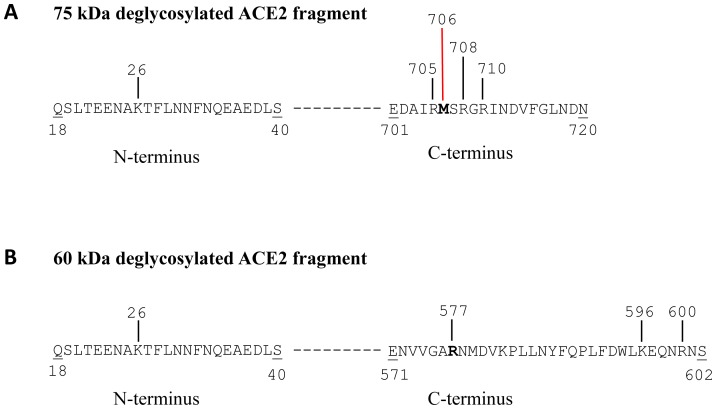
Diagram of 2 shed ACE2 fragments and putative *C*-terminal cleavage sites determined by LC-MS/MS. A) Partial amino acid sequence of 75 kDa shed ACE2 fragment (deglycosylated) is shown, beginning with *N*-terminal amino acid Gln^18^ (Q). The first and last amino acids of the C-terminal and N-terminal fragments are underlined, where numbers below the line indicate their amino acid positions. The dashed line represents amino acids between position 40 and 701 (sequence not shown). The most C-terminal amino acid observed is shown in bold. Vertical black bars represent sites of tryptic cleavage. Red vertical bar is non-tryptic cleavage site at Met^706^ (M), a putative ACE2 cleavage site. B) Partial amino acid sequence of 60 kDa shed ACE2 fragment (deglycosylated) is shown, beginning with *N*-terminal amino acid Gln^18^ (Q). The first and last amino acids of the C-terminal and N-terminal fragments are underlined, where numbers below the line indicate their amino acid positions. The dashed line represents amino acids between position 40 and 571 (sequence not shown). The most C-terminal amino acid observed is shown in bold. Vertical black bars indicate sites of tryptic cleavage, and include Arg^577^ (R) and Lys^596^ (K). Cleavage site for this fragment may occur at or *C*-terminal to Arg^577^.

For the ∼60 kDa mouse ACE2 fragment, trypsin digestion and LC-MS/MS analyses in 5 separate experiments identified peptides corresponding to amino acids 18 to 577 in mouse ACE2, corresponding to a predicted molecular mass of 64.5 kDa (Figure 5 and Table S3). The C-terminal amino acid was Arg^577^, corresponding to a tryptic cleavage site. The cleavage site for this ACE2 fragment is therefore predicted to be located at or C-terminal to Arg^577^ and could occur before Lys^596^, the next C-terminal tryptic cleavage site. In 1 of 6 experiments, LC-MS/MS analysis detected a C-terminal Lys^619^ (a tryptic cleavage site), corresponding to an ACE2 fragment with predicted molecular mass of 69.6 kDa. For this fragment, the cleavage site could lie between Lys^619^ and Arg^644^, since Arg^644^ is the next C-terminal site accessible to tryptic digestion. Alternative proteases (chymotrypsin and glutamyl endopeptidase) were used, but peptides representing the region of interest between Arg^577^ and Lys^596^ were not identified with this strategy (data not shown). A summary of the sequence data for the 2 main ACE2 shed fragments (deglycosylated 75 kDa and 60 kDa fragments) identified by LC-MS/MS is shown in Figure 6.

### Effect of Ang II, high D-glucose and ADAM17 on ACE2 shedding

**Figure 7:**
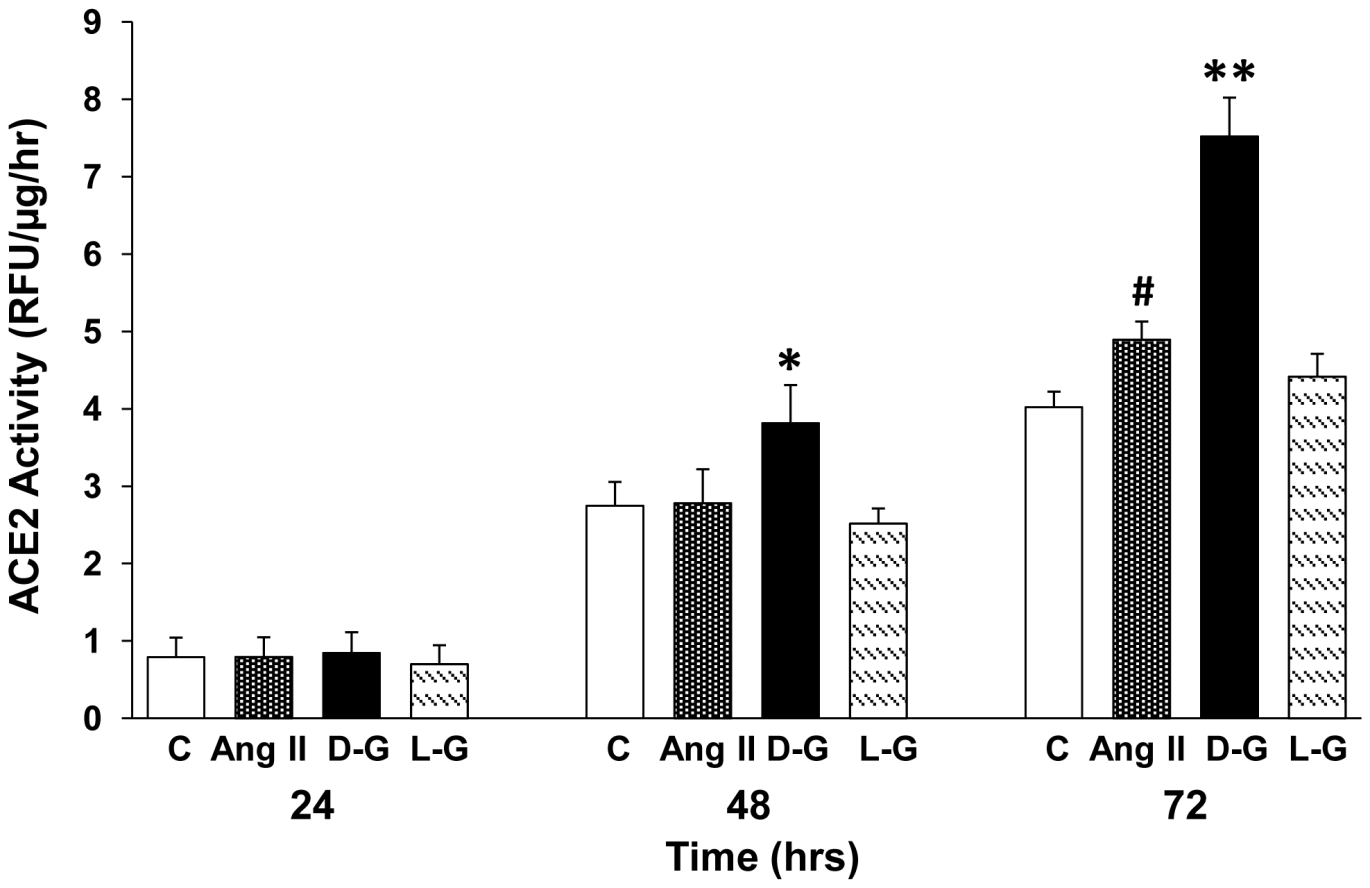
ACE2 shedding is stimulated by high D-glucose or Ang II in mouse PT cells. Primary cultures of mouse PT cells were incubated for 24, 48 and 72(C, 7.8 mM D-glucose), with Ang II (10^−7^ M) or in high D-glucose (D-G, 25 mM) media. As a control for osmolality, some cells were incubated with L-glucose (L-G, 25 mM). ACE2 activity in the media was assayed at each time point. *p<0.001 vs L-G, p<0.004 vs C, p<0.025 vs Ang II, all at 48 hrs, n = 7–9. **p<0.001 vs all 3 other groups at 72 hrs, n = 14–18. ^#^p< 0.04 vs C at 72 hrs, n = 14–18.

Incubation of mouse PT cells with Ang II (10^−7^ M) caused an increase in ACE2 activity in the cell media, which reached statistical significance after 72 hrs (Figure 7). Lower concentrations of Ang II (10^−10^–10^−8^ M) had no significant effect on ACE2 activity in the cell media (n = 4–10; Figure S2). The stimulatory effect of Ang II on ACE2 activity in the culture media was blocked by co-incubation with losartan (10^−5^ M) suggesting that binding of Ang II to PT AT_1_ receptors is required to mediate this effect (Figure S3).

Incubation of cells in high concentrations of D-glucose (25 mM) significantly increased ACE2 activity in the media, with a near doubling at 72 hrs (control [7.8 mM D-glucose]: 4.02±0.20 RFU/µg/hr vs high D-glucose: 7.52±0.50 RFU/µg/hr; P<0.001; n = 14–18). As an osmotic control, incubation of cells in L-glucose (25 mM) had no effect on ACE2 activity in the media. Furthermore, addition of D-glucose (25 mM) to media without cell incubation had no effect on ACE2 activity (n = 3, not shown). Incubation of PT cells in high D-glucose (25 mM) for 72 hrs did not significantly affect ACE2 activity in the cell membrane fraction, compared to cells in 7.8 mM D-glucose (not shown, n = 8).

**Figure 8:**
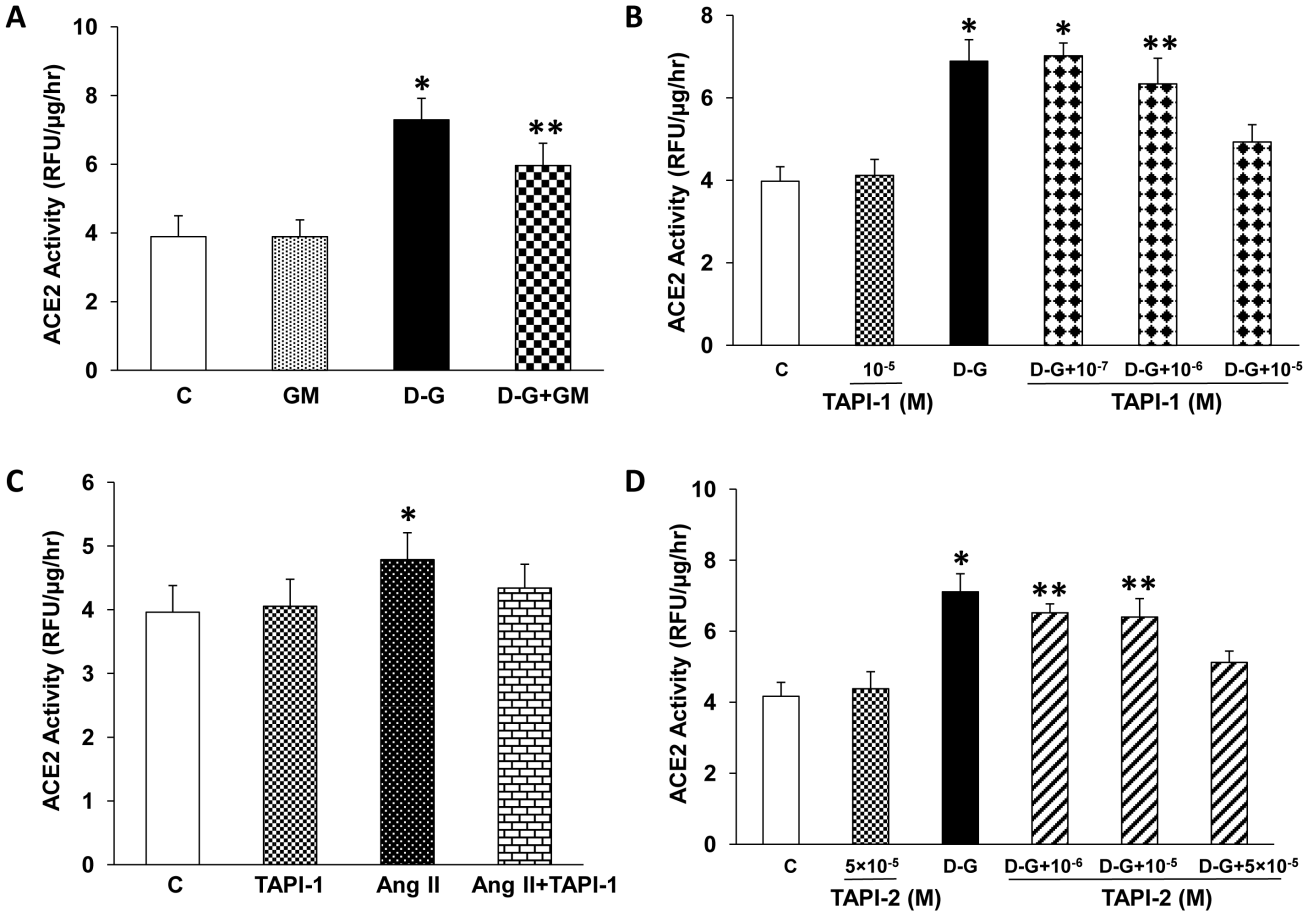
Role of matrix metalloproteinases (MMP) and ADAM17 in high glucose- or Ang II-stimulated ACE2 shedding. (A) Effect of MMP inhibitor GM6001 on ACE2 activity in the media. Mouse PT cells were incubated for 72 hrs in high D-glucose (D–G, 25 mM) media in the presence or absence of GM6001 (GM, 5×10^−5^ M). *p<0.001 vs C and GM, **p<0.025 vs C and GM, n = 5. (B) Effect of the ADAM17 inhibitor TAPI-1 on high glucose-stimulated ACE2 activity in the media. Mouse PT cells were incubated for 72 hrs in high D-glucose (D–G, 25 mM) media in the presence or absence of TAPI-1 (10^−7^–10^−5^ M). *p<0.001 vs C, TAPI-1 10^−5^ M, and D-G+TAPI-1 10^−5^ M. **p<0.001 vs C and TAPI-1 10^−5^ M, p>0.05 vs D-G,D-G+TAPI-1 10^−7^ M and D-G+TAPI-1 10^−5^ M. n = 4–9. (C) Effect of TAPI-1 on Ang II-stimulated ACE2 activity in the media. Mouse PT cells were incubated for 72 hrs with Ang II (10^−7^ M) in the presence or absence of TAPI-1 (10^−5^ M). *p<0.015 vs C, p<0.03 vs TAPI-1, n = 8. (D) Effect of TAPI-2 on high D-glucose-stimulated ACE2 activity in the media. Mouse PT cells were incubated for 72 hrs in high D-glucose (D-G, 25 mM) media in the presence or absence of TAPI-2 (10^−6^– 5×10^−5^ M). *p<0.001 vs C and TAPI-2 (5×10^−5^ M), p<0.015 vs D-G+TAPI-2 (5×10^−5^ M). **p<0.005 vs C, p<0.015 vs TAPI-2 (5×10^−5^ M), p>0.05 vs D-G and D-G+TAPI-2 (5×10^−5^ M). n = 4.

The generalized inhibitor of matrix metalloproteinases GM6001 (5×10^−5^ M) had no significant effect on constitutive ACE2 shedding from PT cells into the media, or on high glucose-stimulated shedding, as determined by ACE2 activity assay (Figure 8A). Lower or higher concentrations of GM6001 (2.5×10^−5^ M, 10^−4^ M) did not affect constitutive or high glucose-inducd ACE2 shedding (data not shown; n = 3). To determine the role of ADAM17 in the stimulation of ACE2 activity in the media induced by high D-glucose, mouse PT cells were pre-incubated with the inhibitor of ADAM17, TAPI-1. TAPI-1 (10^−5^ M) had no significant effect on the constitutive shedding of ACE2 activity into the media (Figure 8B). However, TAPI-1 caused a dose-dependent inhibition of high D-glucose-induced ACE2 shedding, with significant inhibition at 10^−5^ M (control: 3.98±0.35 RFU/µg/hr vs high D-glucose: 6.89±0.52 RFU/µg/hr vs high D-glucose + TAPI-1: 4.93±0.42 RFU/µg/hr; n = 9). TAPI-1 (10^−5^ M) also partly inhibited the small but significant increase in ACE2 activity in the media induced by Ang II (10^−7^ M) (Figure 8C).

To further study the role of ADAM17 in mediating high D-glucose-induced ACE2 shedding, experiments were performed with the ADAM17 inhibitor TAPI-2, at concentrations as described [25]. While TAPI-2 (5×10^−5^ M) had no effect on constitutive ACE2 shedding, it caused a significant inhibition of high D-glucose-induced shedding (Figure 8D). Lower concentrations of TAPI-2 (10^−6^ M, 10^−5^ M) did not significantly block high D-glucose-induced ACE2 shedding.

**Figure 9:**
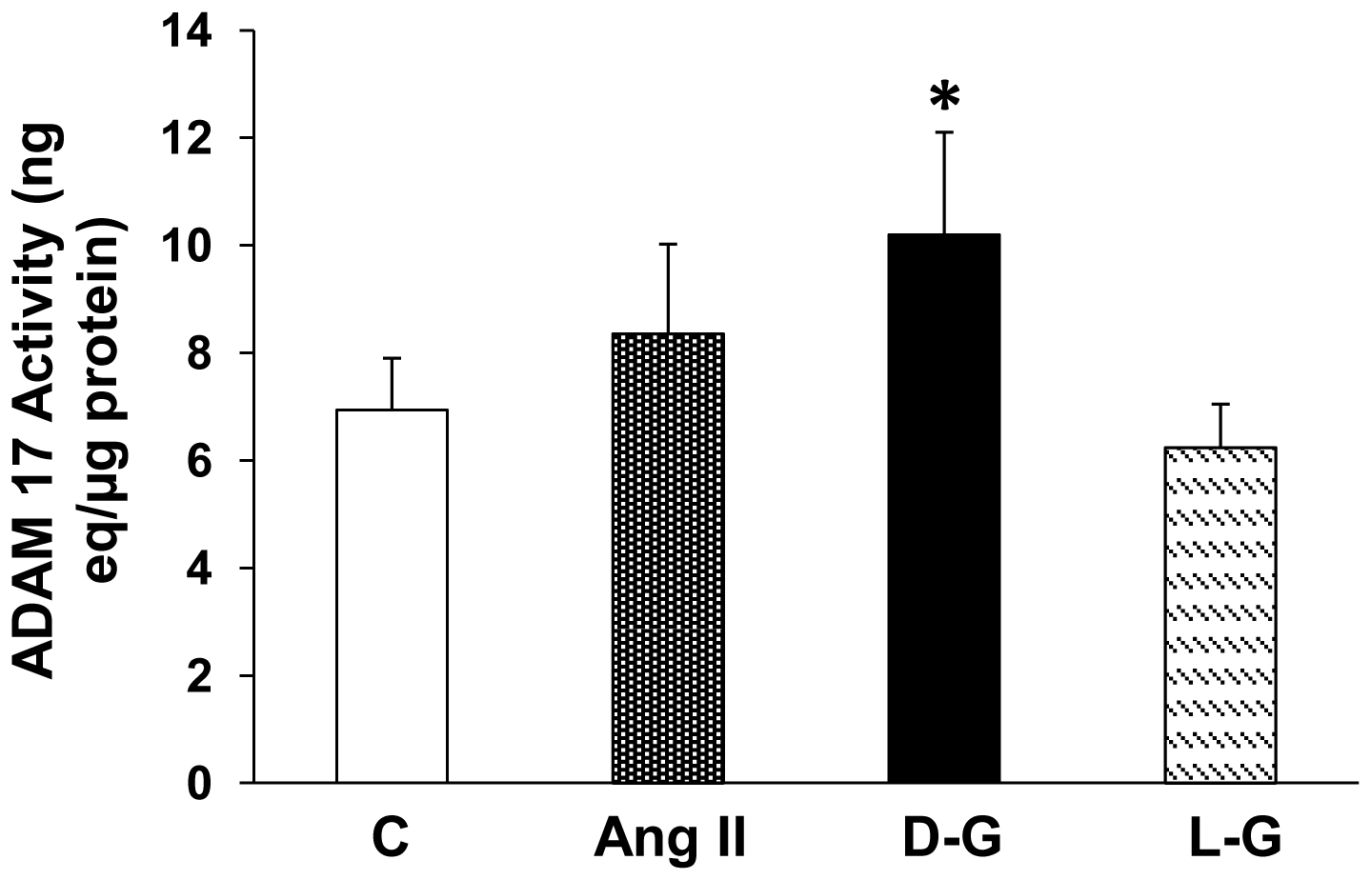
Effect of high D-glucose or Ang II on ADAM17 activity in cell lysates. Mouse PT cells were incubated for 72(10^−7^ M) or in high D-glucose (D-G, 25 mM) media. *p<0.05 vs C, n = 7-10.

After 72 hrs incubation in high D-glucose (25 mM), cell-associated activity of ADAM17 was significantly increased, compared to control cells incubated in 7.8 mM D-glucose or in 25 mM L-glucose (Figure 9).

**Figure 10:**
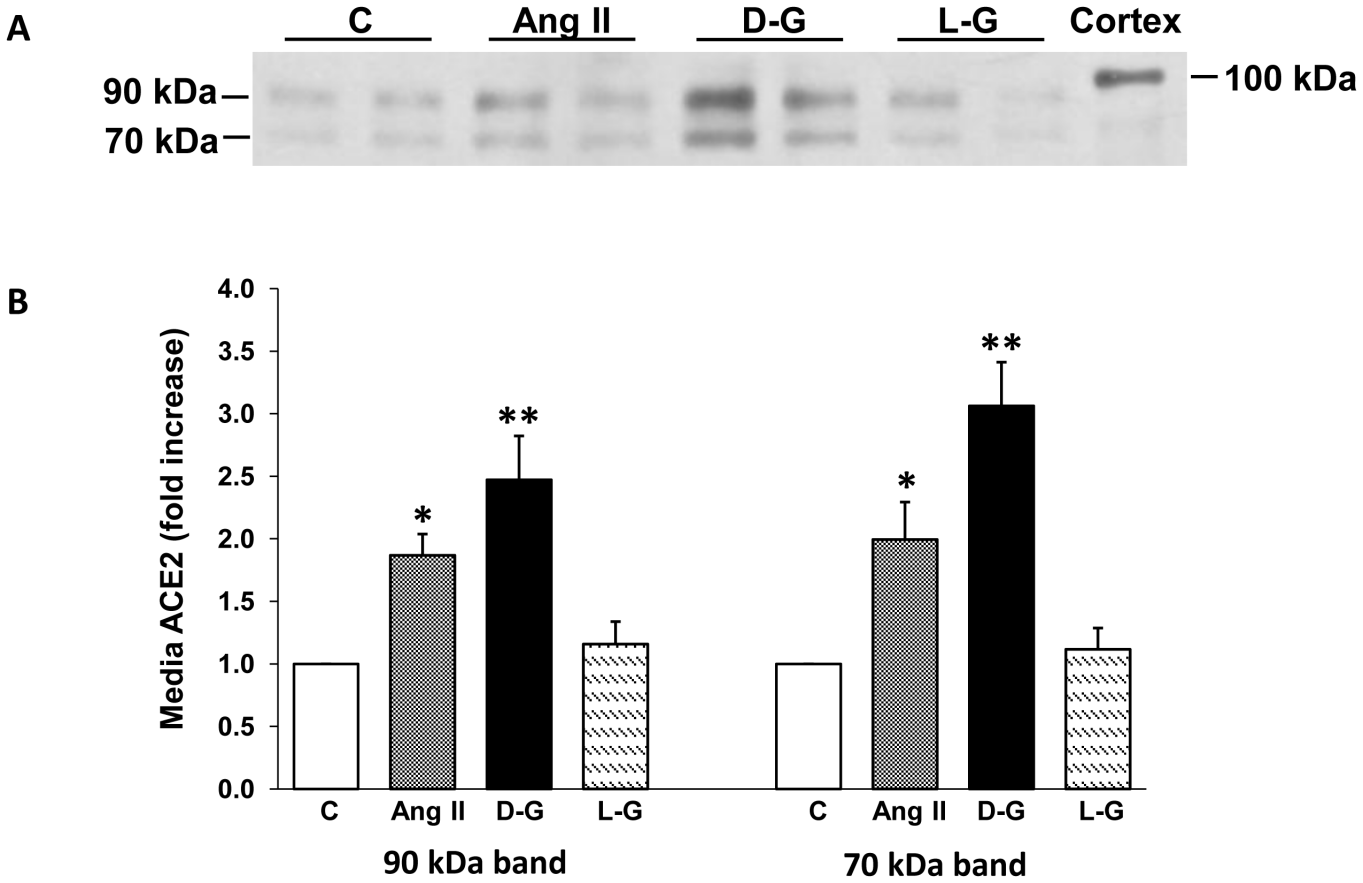
Immunoblot analysis of ACE2 protein in the media from high glucose- or Ang II-stimulated mouse PT cells. (A) Mouse PT cells were incubated for 72 hrs in normal media (C, 7.8 mM D-glucose), with or without Ang II (10^−7^ M), high D-glucose (D-G, 25 mM), or high L-glucose (25 mM). Above graph is representative immunoblot for ACE2 in the media, showing bands at ∼90 kDa and ∼70 kDa. (B) Graphical representation of densitometry analysis of two ACE2 bands on immunoblots. For the ∼90 kDa band, *p<0.05 vs C, **p<0.001 vs C, **p<0.003 vs L-G; n = 5. For the ∼70 kDa band, *p<0.04 vs C; **p<0.001 vs C or L-G, **p<0.03 vs Ang II; n = 5.

To determine if the stimulatory effects of high D-glucose or Ang II on ACE2 activity in cell media were accompanied by increased shedding of ACE2 fragments, immunoblots were performed on media from mouse PT cells. Constitutive shedding of the ∼90 kDa and ∼70 kDa ACE2 fragments was evident in unconcentrated media from unstimulated mouse PT cells after 72 hrs in culture. Incubation of the cells for 72 hrs with either Ang II (10^−7^ M) or high D-glucose (25 mM) significantly increased the intensity of both bands on immunoblots, compared to 7.8 mM D-glucose, or 25 mM L-glucose (Figure 10). Besides the ∼90 kDa and ∼70 kDa ACE2 fragments, no additional bands were detected on ACE2 immunoblots of media derived from cells exposed to high D-glucose or Ang II.

After immunoprecipitation with an ACE2 antibody, LC-MS/MS analysis of the ∼75 kDa and ∼60 kDa deglycosylated peptide fragments isolated from high D-glucose media after 72 hrs confirmed that the 2 fragments were derived from ACE2. In 3 separate LC-MS/MS analyses, the C-terminus of the ∼75 kDa fragment isolated from high glucose media corresponded to Arg^705^ in ACE2. For the ∼60 kDa fragment, 4 out of 5 LC-MS/MS analyses demonstrated a tryptic fragment with C-terminus as Arg^577^, consistent with the fragment isolated in normal (7.8 mM) glucose media. One out of 5 analyses revealed an ACE2 fragment with C-terminus as Lys^619^.

## Discussion

The present studies are the first to demonstrate shedding of active fragments of ACE2 from kidney PT cells, a finding that could at least partly account for the presence of soluble ACE2 in mouse [15], [19] and human urine [14], [16]–[18]. We also show that high glucose, and to a lesser extent Ang II, stimulate ACE2 release from PT cells, which may contribute mechanistically to the increased urinary levels of ACE2 observed in experimental and human diabetes [8], [15], [17], [19]. Inhibition of ADAM17 blocked glucose-stimulated shedding of ACE2, and partly inhibited Ang II-induced shedding, but had no impact on constitutive release. Ectodomain shedding of ACE2 was associated with release of 2 soluble glycosylated peptides of approximate molecular masses 90 kDa and 70 kDa. These fragments were deglycosylated and subjected to LC-MS/MS analysis, which revealed a C-terminal Met^706^ in the long fragment, and a C-terminal Arg^577^ in the shorter fragment, with the predicted molecular masses of both fragments corresponding to their approximate sizes (75 kDa and 60 kDa, respectively) on 2-D gels.

ACE2 is an 805-amino acid type I transmembrane glycoprotein that is localized mainly to the apical membrane in polarized epithelial cells [14]. As a terminal carboxypeptidase, ACE2 consists of a *N*-glycosylated N-terminal ectodomain that contains the active site (amino acids 1-740), a hydrophobic transmembrane region (amino acids 741–762), and a short C-terminal cytoplasmic tail (amino acids 763–805) [13]. Cell surface abundance of transmembrane proteins may decrease as a result of ectodomain shedding, which may be mediated by proteolytic cleavage at specific amino acid sites. Constitutive ectodomain shedding of soluble ACE2 has been demonstrated in a variety of cultured cells, including ACE2-transfected HEK293 cells and endogenous ACE2-expressing Huh7 cells [11], human airway epithelial cells [13], ACE2-transfected Chinese Hamster Ovary (CHO) cells [12] and mouse embryonic fibroblasts infected with adenovirus encoding ACE2 [12]. We observed a time-dependent increase in ACE2 activity in the media of unstimulated mouse PT cells, associated with the appearance of two ACE2 fragments on immunoblots. These data indicate that ACE2 is constitutively shed from mouse PT cells, and indeed we also observed constitutive shedding from PT cells derived from ACE2 KO mice that had been transiently transfected with a human ACE2 cDNA expression vector, by immunoblot and enzymatic activity assay.

Like ACE2, ADAM17 is a transmembrane metalloproteinase, which acts as a “sheddase” and cleaves cell surface proteins, including tumour necrosis factor-α, the type II interleukin-1 receptor, and transforming growth factor-α [12], [26], [27]. Lambert et al. first reported that ADAM17 mediated the proteolytic cleavage of human ACE2 in HEK293 and endogenous ACE2 in Huh7 cells, with release of 2 soluble glycosylated fragments of size 105 kDa and 95 kDa [11]. Phorbol ester-stimulated release of ACE2 was blocked by inhibition of ADAM17 with TAPI-1, and by generalized metalloprotease inhibition with the hydroxymate-based inhibitor GM6001 [11]. TAPI-1 has also been shown to significantly inhibit phorbol ester-stimulated ACE2 shedding in ACE2-transfected CHO cells, with little effect on constitutive shedding [28]. In ACE2-transfected HEK293 cells, basal shedding of ACE2 was not significantly affected by either TAPI-1 or GM6001 [11]. By contrast, constitutive shedding of soluble ACE2 in human airway epithelial cells is reduced by ADAM17 inhibition, as is phorbol ester-stimulated release, suggesting a prominent role for ADAM17 in ACE2 cleavage in these cells [13]. Interestingly, in our studies TAPI-1 completely blocked high glucose-stimulated shedding of ACE2 from PT cells, and caused a partial inhibition of Ang II-stimulated ACE2 shedding. TAPI-1 had no effect on constitutive ACE2 release. The ADAM17 inhibitor TAPI-2 also inhibited high glucose-stimulated ACE2 shedding from PT cells. In contrast, generalized metalloproteinase inhibition with GM6001 had no impact on constitutive ACE2 shedding, and did not significantly inhibit high glucose-stimulated shedding. Our data therefore suggest that basal ACE2 shedding in PT cells is mediated by other protease(s), or by a relative lack of tissue inhibitors of metalloproteinases (TIMPs) [11]. Indeed, since the inhibitors used in this study are not highly selective, our data support a possible role for ADAMs and metalloproteinases in the shedding of ACE2 from PT cells.

We identified 2 major cleaved fragments of ACE2 by immunoblots in media from PT cells. The glycosylated fragments ran at ∼90 kDa and ∼70 kDa on gels, and collectively, they retained ACE2 enzymatic activity. Although we did not determine the differential enzymatic activity of the fragments, the presence of the ACE2 ectodomain in each sequence predicts retention of ACE2 activity. In support of this hypothesis, Iwata et al. demonstrated that deglycosylated soluble shed fragments of human ACE2 at ∼80 kDa and ∼70 kDa each retained equivalent ACE2 enzymatic activities [12].

Our results are not the first to suggest that ACE2 fragments may be shed from renal tubular cells. Wysocki et al. detected ACE2 in db/db diabetic mouse urine samples by immunoblot, at molecular sizes of 110 kDa and 75 kDa, with the larger band consistent with the size of full-length ACE2 [15]. Chodavarapu et al. reported ACE2 fragments of molecular size 90 kDa and 70 kDa in urines from db/db mice, with the larger band corresponding to that observed on immunblots of kidney lysates [19]. Deglycosylation studies and further characterization of these urinary ACE2 fragments are required to determine if they correspond to the mouse PT ACE2 fragments identified in the current studies.

The present studies showed that high glucose and Ang II stimulated the shedding of 2 soluble ACE2 fragments of equivalent size to those fragments released by unstimulated cells. ACE2 fragments were identified beginning at Gln^18^, consistent with the presence of a 17-amino acid signal peptide sequence at the *N*-terminus [2]. In unstimulated cells, the long soluble fragment was shown to contain a Met^706^ at the C-terminal position (not subject to tryptic digestion). This site was found in one experiment, while 3 other, separate samples identified a trypsin-sensitive Arg^705^ at the C-terminus. Similarly, in stimulated cells, the C-terminal amino acid of the long fragment was identified as Arg^705^ in 3 separate experiments. Using a series of synthesized peptides corresponding to the extracellular juxtamembrane region of human ACE2, Lai et al. reported that Arg^708^ and Arg^710^ may play roles in site recognition for ACE2 shedding mediated by ADAM17 [28]. A putative cleavage site for ADAM17-mediated shedding was identified at the bond Arg^708^-Ser^709^ in human ACE2 [28]. While the mouse ACE2 amino acid sequence contains conserved residues at Arg^708^ and Arg^710^, Arg^708^ is followed by Gly^709^, suggesting that species-specific cleavage sites may exist. In this regard, further studies directed at LC-MS/MS analysis of ACE2 fragments in media from ACE2 KO cells stably transfected with the human ACE2 cDNA vector could provide supportive information. Our data support Met^706^ as a potential cleavage site, although we cannot rule out the possibility for other cleavage sites in proximity (including Arg^705^), since these would generate fragments that might not be distinguishable on gel electrophoresis.

An Arg^577^ was identified as the C-terminal amino acid for the shorter 60 kDa deglycosylated ACE2 fragment in both unstimulated and stimulated PT cells. Since this represents a trypsin-sensitive cleavage site, it is possible that the ACE2 cleavage site for this fragment may be downstream of Arg^577^, perhaps at a position up to Lys^596^, the next tryptic cleavage site. ADAM17 has some selectivity for aliphatic hydrophobic residues at the P1′ position (immediately downstream of the cleavage site), and the P1′ amino acid for Arg^577^ is Asn^578^ (a hydrophilic residue), although considerable variability exists in substrate recognition sites [29]. Jia et al. have reported that mutation of Leu^584^ in human ACE2 to Ala^584^ prevents shedding, suggesting that this represents a sheddase “recognition motif” [13]. Whether this position, conserved in mouse ACE2, is a putative cleavage site for the shorter ACE2 fragment remains unclear.

An ACE2 peptide fragment with C-terminus as Lys^619^ (a tryptic cleavage site) was identified by LC-MS/MS of the immunoprecipitated shorter fragment in 1 out of 6 experiments for constitutive shedding, and 1 out of 5 experiments for glucose-stimulated shedding. The significance of this finding is uncertain, although this peptide could represent an additional fragment of ACE2 that is constitutively shed, albeit at lower levels compared to the ∼75 kDa and ∼60 kDa fragments.

The biologic relevance of ACE2 shedding from PT cells is unknown. Mice with diabetes exhibit increased kidney expression of ADAM17 and ACE2, associated with high levels of urinary ACE2 activity and shedding of ACE2 fragments [15], [19]. In humans, increased urinary ACE2 levels are associated with a higher risk of type 2 diabetes, and are predictive of microalbuminuria, suggesting that urinary ACE2 may be a biomarker of early diabetic nephropathy [18]. Consistent with these observations, in our studies high glucose stimulated PT cell ADAM17 activity, and led to increased ACE2 shedding. By contrast, Ang II did not significantly increase ADAM17 activity, although TAPI-1 partly inhibited Ang II-stimulated ACE2 shedding. In this regard, the effect of Ang II on ACE2 shedding was relatively minor, and any increases in ADAM17 activity induced by Ang II may have been below the limits of detectability in the enzyme assay. Taken together, our results suggest that increases in luminal ACE2 activity occur in the diabetic kidney, stimulated by high glucose and Ang II. Shed ACE2 could serve to dampen levels of Ang II in the PT lumen, and increase Ang-(1-7) production. On the other hand, ectodomain shedding of ACE has been shown to occur in polarized renal epithelial cells [14], and *N*-domain ACE fragments are present in human urine and may associate with hypertension [30]. Although we did not study PT ACE shedding, it is intriguing to speculate that the relative balance between shed ACE2 and ACE fragments could regulate local levels of Ang II and Ang-(1-7) in the urinary space.

In summary, mouse PT cells constitutively shed 2 enzymatically active glycosylated fragments of ACE2, of molecular masses ∼90 kDa and 70 kDa. The release of these fragments is stimulated by high glucose and Ang II, in a manner dependent on ADAM17. In diabetic nephropathy, enhanced PT shedding of ACE2 fragments via ADAM17 could increase Ang II degrading capacity in the urine, and could serve as a biomarker of early kidney injury.

## Supporting Information

Figure S1 **Time course of ACE2 activity assay in cell culture media.** ACE2 activity (RFU) in culture media from mouse PT cells (5, 10 and 15 µL) was measured at 2, 6, 16 and 24 hrs after incubation with substrate. A highly linear relationship exists between the incubation time and the RFUs for different volumes of cell culture medium (p<0.003 for 5 µL and 10 µL; p<0.005 for 15 µL; n = 3).
(TIF)

Figure S2 **Dose-dependent effects of Ang II on ACE2 activity in the media from PT cells in culture.** Primary cultures of mouse PT cells were incubated for 72 hrs with varying concentrations of Ang II (10^−10^–10^−7^ M). *p<0.05 vs C, n = 4–10.
(TIF)

Figure S3 **Effect of AT_1_ receptor antagonist losartan on Ang II-stimulated ACE2 activity in media from PT cells.** Mouse PT cells were incubated for 72 hrs with Ang II (10^−7^ M) in the presence or absence of losartan (Los, 10^−5^ M). *p<0.05 vs all other groups, n = 9–10.
(TIF)

Table S1 **Effect of MLN-4760 on ACE2 activity in PT cell culture media.**
(DOC)

Table S2 **ACE2 peptides identified by LC-MS/MS in the 75 kDa protein band.**
(DOC)

Table S3 **ACE2 peptides identified by LC-MS/MS in the 60 kDa protein band.**
(DOC)

## References

[1] Donoghue M, Hsieh F, Baronas E, Godbout K, Gosselin M, et al (2000) A novel angiotensin-converting enzyme-related carboxypeptidase (ACE2) converts angiotensin I to angiotensin 1-9. Circ Res 87: E1–9. 10969042 10.1161/01.res.87.5.e1

[2] Tipnis SR, Hooper NM, Hyde R, Karran E, Christie G, et al (2000) A human homolog of angiotensin-converting enzyme. Cloning and functional expression as a captopril-insensitive carboxypeptidase. J Biol Chem 275: 33238–33243. 10924499 10.1074/jbc.M002615200

[3] Ye M, Wysocki J, Naaz P, Salabat MR, LaPointe MS, et al (2004) Increased ACE 2 and decreased ACE protein in renal tubules from diabetic mice: a renoprotective combination? Hypertension 43: 1120–1125. 15078862 10.1161/01.HYP.0000126192.27644.76

[4] Li N, Zimpelmann J, Cheng K, Wilkins JA, Burns KD (2005) The role of angiotensin converting enzyme 2 in the generation of angiotensin 1-7 by rat proximal tubules. Am J Physiol Renal Physiol 288: F353–362. 15467007 10.1152/ajprenal.00144.2004

[5] Oudit GY, Herzenberg AM, Kassiri Z, Wong D, Reich H, et al (2006) Loss of angiotensin-converting enzyme-2 leads to the late development of angiotensin II-dependent glomerulosclerosis. Am J Pathol 168: 1808–1820. 16723697 10.2353/ajpath.2006.051091 PMC1606622

[6] Wong DW, Oudit GY, Reich H, Kassiri Z, Zhou J, et al (2007) Loss of angiotensin-converting enzyme-2 (Ace2) accelerates diabetic kidney injury. Am J Pathol 171: 438–451. 17600118 10.2353/ajpath.2007.060977 PMC1934545

[7] Oudit GY, Liu GC, Zhong J, Basu R, Chow FL, et al (2010) Human recombinant ACE2 reduces the progression of diabetic nephropathy. Diabetes 59: 529–538. 19934006 10.2337/db09-1218 PMC2809962

[8] Nadarajah R, Milagres R, Dilauro M, Gutsol A, Xiao F, et al (2012) Podocyte-specific overexpression of human angiotensin-converting enzyme 2 attenuates diabetic nephropathy in mice. Kidney Int 82: 292–303. 22475818 10.1038/ki.2012.83 PMC3410252

[9] Reich HN, Oudit GY, Penninger JM, Scholey JW, Herzenberg AM (2008) Decreased glomerular and tubular expression of ACE2 in patients with type 2 diabetes and kidney disease. Kidney Int 74: 1610–1616. 19034303 10.1038/ki.2008.497

[10] Ye M, Wysocki J, William J, Soler MJ, Cokic I, et al (2006) Glomerular localization and expression of Angiotensin-converting enzyme 2 and Angiotensin-converting enzyme: implications for albuminuria in diabetes. J Am Soc Nephrol 17: 3067–3075. 17021266 10.1681/ASN.2006050423

[11] Lambert DW, Yarski M, Warner FJ, Thornhill P, Parkin ET, et al (2005) Tumor necrosis factor-alpha convertase (ADAM17) mediates regulated ectodomain shedding of the severe-acute respiratory syndrome-coronavirus (SARS-CoV) receptor, angiotensin-converting enzyme-2 (ACE2). J Biol Chem 280: 30113–30119. 15983030 10.1074/jbc.M505111200 PMC8062222

[12] Iwata M, Silva Enciso JE, Greenberg BH (2009) Selective and specific regulation of ectodomain shedding of angiotensin-converting enzyme 2 by tumor necrosis factor alpha-converting enzyme. Am J Physiol Cell Physiol 297: C1318–1329. 19759332 10.1152/ajpcell.00036.2009

[13] Jia HP, Look DC, Tan P, Shi L, Hickey M, et al (2009) Ectodomain shedding of angiotensin converting enzyme 2 in human airway epithelia. Am J Physiol Lung Cell Mol Physiol 297: L84–96. 19411314 10.1152/ajplung.00071.2009 PMC2711803

[14] Warner FJ, Lew RA, Smith AI, Lambert DW, Hooper NM, et al (2005) Angiotensin-converting enzyme 2 (ACE2), but not ACE, is preferentially localized to the apical surface of polarized kidney cells. J Biol Chem 280: 39353–39362. 16166094 10.1074/jbc.M508914200

[15] Wysocki J, Garcia-Halpin L, Ye M, Maier C, Sowers K, et al (2013) Regulation of Urinary ACE2 in Diabetic Mice. Am J Physiol Renal Physiol 305: F600–F611. 23761674 10.1152/ajprenal.00600.2012 PMC3891267

[16] Mizuiri S, Aoki T, Hemmi H, Arita M, Sakai K, et al (2011) Urinary angiotensin-converting enzyme 2 in patients with CKD. Nephrology (Carlton) 16: 567–572. 21457402 10.1111/j.1440-1797.2011.01467.x

[17] Xiao F, Hiremath S, Knoll G, Zimpelmann J, Srivaratharajah K, et al (2012) Increased urinary angiotensin-converting enzyme 2 in renal transplant patients with diabetes. PLoS One 7: e37649. 22629438 10.1371/journal.pone.0037649 PMC3358292

[18] Park SE, Kim WJ, Park SW, Park JW, Lee N, et al (2013) High urinary ACE2 concentrations are associated with severity of glucose intolerance and microalbuminuria. Eur J Endocrinol 168: 203–210. 23144053 10.1530/EJE-12-0782

[19] Chodavarapu H, Grobe N, Somineni HK, Salem ES, Madhu M, et al (2013) Rosiglitazone treatment of type 2 diabetic db/db mice attenuates urinary albumin and angiotensin converting enzyme 2 excretion. PLoS One 8: e62833. 23646149 10.1371/journal.pone.0062833 PMC3639987

[20] Vinay P, Gougoux A, Lemieux G (1981) Isolation of a pure suspension of rat proximal tubules. Am J Physiol 241: F403–411. 6119031 10.1152/ajprenal.1981.241.4.F403

[21] Ye M, Wysocki J, Gonzalez-Pacheco FR, Salem M, Evora K, et al (2012) Murine recombinant angiotensin-converting enzyme 2: effect on angiotensin II-dependent hypertension and distinctive angiotensin-converting enzyme 2 inhibitor characteristics on rodent and human angiotensin-converting enzyme 2. Hypertension 60: 730–740. 22777933 10.1161/HYPERTENSIONAHA.112.198622 PMC3426447

[22] Kassiri Z, Oudit GY, Kandalam V, Awad A, Wang X, et al (2009) Loss of TIMP3 enhances interstitial nephritis and fibrosis. J Am Soc Nephrol 20: 1223–1235. 19406980 10.1681/ASN.2008050492 PMC2689897

[23] Dilauro M, Zimpelmann J, Robertson SJ, Genest D, Burns KD (2010) Effect of ACE2 and angiotensin-(1-7) in a mouse model of early chronic kidney disease. Am J Physiol Renal Physiol 298: F1523–1532. 20357030 10.1152/ajprenal.00426.2009

[24] Shevchenko A, Tomas H, Havlis J, Olsen JV, Mann M (2006) In-gel digestion for mass spectrometric characterization of proteins and proteomes. Nat Protoc 1: 2856–2860. 17406544 10.1038/nprot.2006.468

[25] Fréour T, Jarry A, Bach-Ngohou K, Dejoie T, Bou-Hanna C, et al (2009) TACE inhibition amplifies TNF-alpha-mediated colonic epithelial barrier disruption. Int J Mol Med 23: 41–48. 19082505

[26] Reddy P, Slack JL, Davis R, Cerretti DP, Kozlosky CJ, et al (2000) Functional analysis of the domain structure of tumor necrosis factor-alpha converting enzyme. J Biol Chem 275: 14608–14614. 10799547 10.1074/jbc.275.19.14608

[27] Fan H, Turck CW, Derynck R (2003) Characterization of growth factor-induced serine phosphorylation of tumor necrosis factor-alpha converting enzyme and of an alternatively translated polypeptide. J Biol Chem 278: 18617–18627. 12621058 10.1074/jbc.M300331200

[28] Lai ZW, Hanchapola I, Steer DL, Smith AI (2011) Angiotensin-converting enzyme 2 ectodomain shedding cleavage-site identification: determinants and constraints. Biochemistry 50: 5182–5194. 21563828 10.1021/bi200525y

[29] Caescu CI, Jeschke GR, Turk BE (2009) Active-site determinants of substrate recognition by the metalloproteinases TACE and ADAM10. Biochem J 424: 79–88. 19715556 10.1042/BJ20090549 PMC2774824

[30] Maluf-Meiken LC, Fernandes FB, Aragão DS, Ronchi FA, Andrade MC, et al (2012) N-domain isoform of Angiotensin I converting enzyme as a marker of hypertension: populational study. Int J Hypertens 2012: 58170. 10.1155/2012/581780 PMC3362081 22666552

