# Supplementary figures and images for: Characterization of Angiotensin-Converting Enzyme 2 Ectodomain Shedding from Mouse Proximal Tubular Cells
Source: PLoS One. 2014 Jan 15;9(1):e85958. doi: 10.1371/journal.pone.0085958 (PMC3893316; doi:10.1371/journal.pone.0085958)

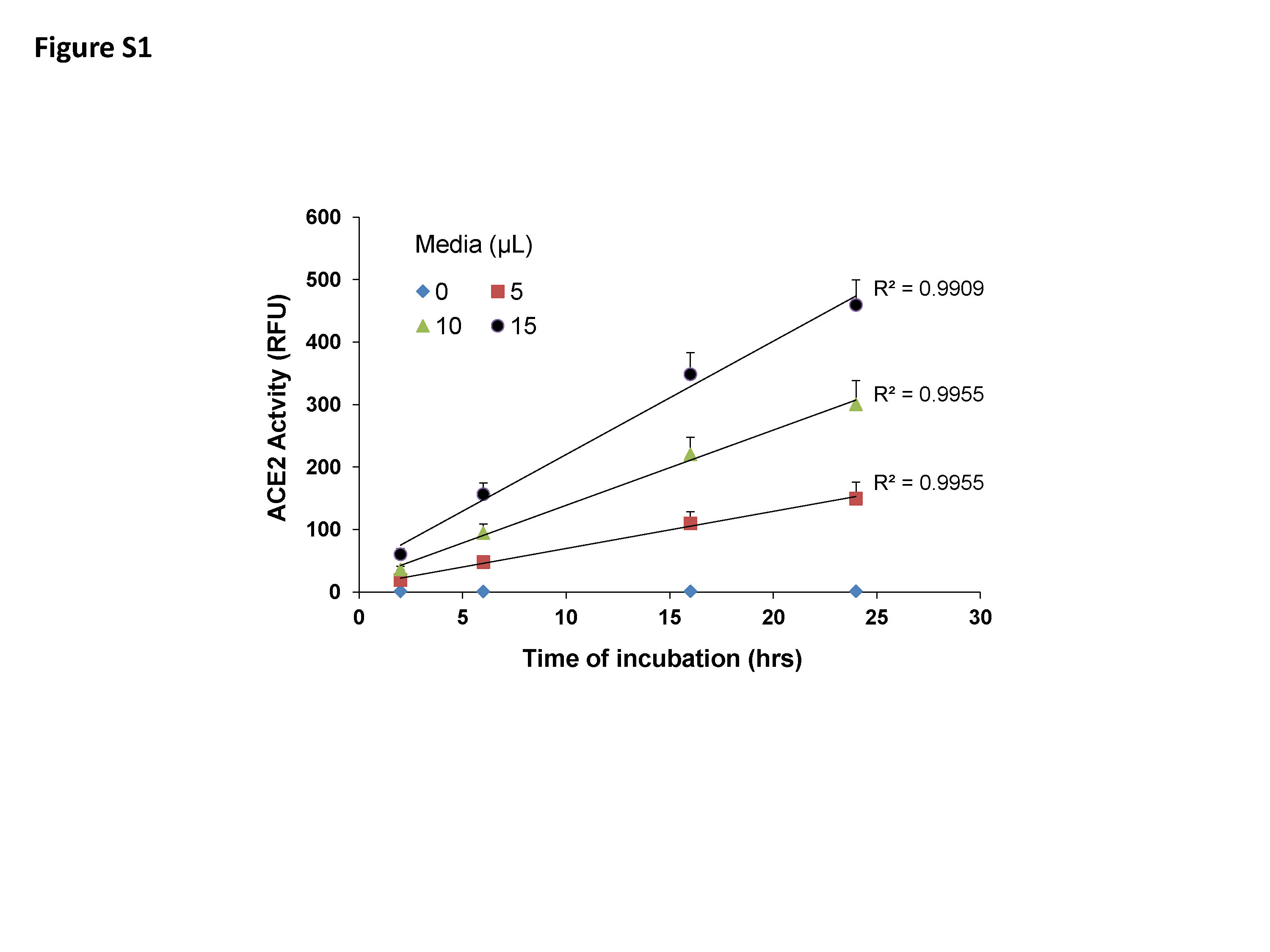

Supplement: Figure S1 — Time course of ACE2 activity assay in cell culture media. ACE2 activity (RFU) in culture media from mouse PT cells (5, 10 and 15 µL) was measured at 2, 6, 16 and 24 hrs after incubation with substrate. A highly linear relationship exists between the incubation time and the RFUs for different volumes of cell culture medium (p<0.003 for 5 µL and 10 µL; p<0.005 for 15 µL; n = 3). (TIF) [file pone.0085958.s001.tif]

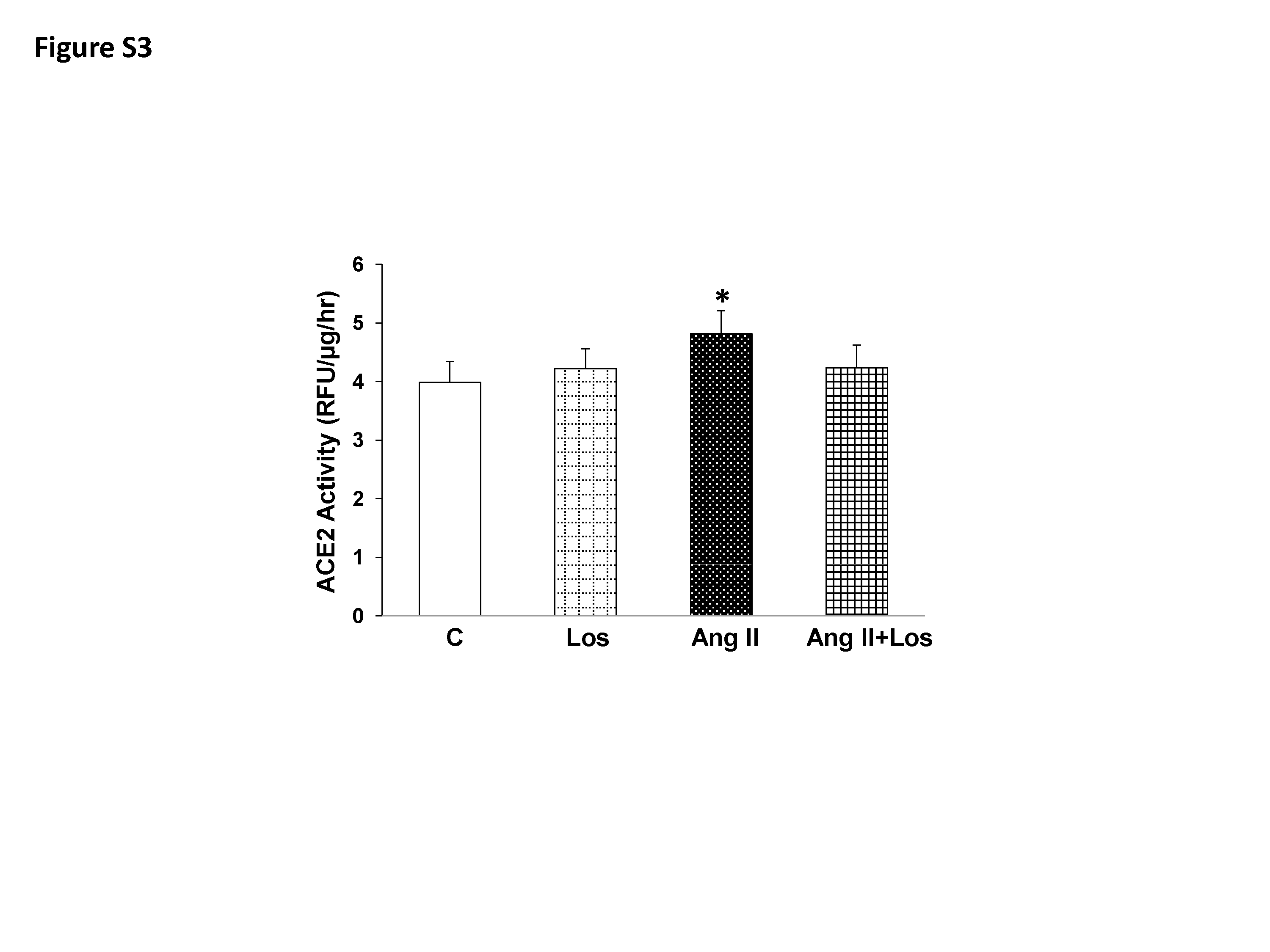

Supplement: Figure S3 — Effect of AT1 receptor antagonist losartan on Ang II-stimulated ACE2 activity in media from PT cells. Mouse PT cells were incubated for 72 hrs with Ang II (10−7 M) in the presence or absence of losartan (Los, 10−5 M). *p<0.05 vs all other groups, n = 9–10. (TIF) [file pone.0085958.s003.tif]
