# Supplementary material for: Characterization of Angiotensin-Converting Enzyme 2 Ectodomain Shedding from Mouse Proximal Tubular Cells
Source: PLoS One. 2014 Jan 15;9(1):e85958. doi: 10.1371/journal.pone.0085958 (PMC3893316; doi:10.1371/journal.pone.0085958)
Supplement: Table S1 — Effect of MLN-4760 on ACE2 activity in PT cell culture media. (DOC) [file pone.0085958.s004.doc]

# Supporting Information

**Table S1. Effect of MLN-4760 on ACE2 activity in cell culture media**

|  | **Control** | **Ang II** | **D-glucose** | **L-Glucose** | **Average** | **S.E.** |
| --- | --- | --- | --- | --- | --- | --- |
| RFU^*^ (without MLN-4760) | 220.08 | 269.08 | 372.08 | 230.08 |  |  |
| RFU (with MLN-4760) | 7.08 | 8.08 | 9.08 | 7.58 |  |  |
| Inhibition (%) | 96.78 | 97.00 | 97.56 | 96.71 | 97.01 | 0.19 |

^*^RFU: Relative Fluorescent Units. Sample raw data are shown from one experiment, to indicate % inhibition by MLN-4760 in the assay. The RFU is an average value of measurements from the media of cell culture dishes, performed in duplicate.
