# Supplementary material for: Characterization of Angiotensin-Converting Enzyme 2 Ectodomain Shedding from Mouse Proximal Tubular Cells
Source: PLoS One. 2014 Jan 15;9(1):e85958. doi: 10.1371/journal.pone.0085958 (PMC3893316; doi:10.1371/journal.pone.0085958)
Supplement: Table S2 — ACE2 peptides identified by LC-MS/MS in the 75 kDa protein band. (DOC) [file pone.0085958.s005.doc]

# Supporting Information

**Table S2. ACE2 peptides identified by LC-MS/MS in the 75 kDa protein band**

| **Position** | **Sequence** | **Ions score*^a^*** | **Observed*^b^*** | **Mr(expt)*^c^*** | **Mr(calc)*^d^*** | **ppm*^e^*** | **Miss*^f^*** |
| --- | --- | --- | --- | --- | --- | --- | --- |
| 18 – 26 | QSLTEENAK | 27 | 501.74 | 1001.47 | 1001.47 | -1 | 0 |
| 61 – 68 | KMSEAAAK | 4 | 426.22 | 850.42 | 850.42 | -1 | 1 |
| 69 – 78 | **WSAFYEEQSK** | 57 | 637.79 | 1273.56 | 1273.56 | 2 | 0 |
| 79 – 94 | **TAQSFSLQEIQTPIIK** | 58 | 902.50 | 1802.98 | 1802.98 | 3 | 0 |
| 79 – 95 | **TAQSFSLQEIQTPIIKR** | 40 | 654.04 | 1959.09 | 1959.08 | 5 | 1 |
| 96 – 112 | **QLQALQQSGSSALSADK** | 88 | 866.45 | 1730.88 | 1730.88 | -1 | 0 |
| 96 – 114 | QLQALQQSGSSALSADKNK | 30 | 658.68 | 1973.03 | 1973.02 | 6 | 1 |
| 115 - 131 | **QLNTILNTMSTIYSTGK** | 60 | 943.00 | 1883.98 | 1883.97 | 5 | 0 |
| 162 - 169 | LWAWEGWR | 30 | 552.28 | 1102.54 | 1102.53 | 2 | 0 |
| 220 - 228 | **NQLIEDVER** | 53 | 558.29 | 1114.56 | 1114.56 | 2 | 0 |
| 307 - 313 | IFQEAEK | 24 | 432.73 | 863.44 | 863.44 | -1 | 0 |
| 331 - 340 | SMLTEPADGR | 25 | 546.75 | 1091.49 | 1091.49 | -1 | 0 |
| 394 - 416 | NGANEGFHEAVGEIMSLSAATPK | 24 | 782.71 | 2345.11 | 2345.10 | 6 | 0 |
| 420 - 441 | **SIGLLPSDFQEDSETEINFLLK** | 65 | 1248.14 | 2494.26 | 2494.25 | 5 | 0 |
| 519 - 534 | **TIYQFQFQEALCQAAK** | 64 | 973.48 | 1944.95 | 1944.94 | 5 | 0 |
| 557 - 568 | **MLSLGNSEPWTK** | 80 | 681.84 | 1361.67 | 1361.66 | 4 | 0 |
| 569 - 577 | **ALENVVGAR** | 64 | 464.76 | 927.52 | 927.51 | 2 | 0 |
| 645 - 652 | **SSVAYAMR** | 61 | 442.72 | 883.42 | 883.42 | -3 | 0 |
| 654 - 671 | YFSIIKNQTVPFLEEDVR | 15 | 733.72 | 2198.14 | 2198.13 | 5 | 1 |
| 679 - 697 | **VSFYFFVTSPQNVSDVIPR** | 102 | 1102.06 | 2202.11 | 2202.10 | 6 | 0 |
| 698 - 705 | **SEVEDAIR** | 35 | 459.73 | 917.44 | 917.45 | -2 | 0 |
| 698 - 706 | **SEVEDAIRM** | 50 | 533.25 | 1064.48 | 1064.48 | 2 | 1 |

**Note**: ACE2 peptide sequences, positions, and matched scores were determined with LC-MS/MS analysis, and statistically significant matched peptide sequences were underlined and shown in bold. The cutoff ions score (p<0.05) was 34, in which individual peptide matches with score above 34 are considered statistically significant. False discovery rate was 3.05%. MS error tolerance used was ±7 ppm.

*^a^* a measure of statistical significance of how well the observed MS/MS spectrum matches to the theoretical spectrum of the stated peptide.

*^b^* experimentally determined mass to charge ratio of the ionized molecule (m/z).

*^c^* experimentally determined mass.

*^d^* theoretical calculated mass of the matched peptide.

*^e^* "parts per million" difference (error) between the experimentally observed mass and the theoretically calculated mass.

*^f^* number of missed cleavage sites.
