# Supplementary material for: Characterization of Angiotensin-Converting Enzyme 2 Ectodomain Shedding from Mouse Proximal Tubular Cells
Source: PLoS One. 2014 Jan 15;9(1):e85958. doi: 10.1371/journal.pone.0085958 (PMC3893316; doi:10.1371/journal.pone.0085958)
Supplement: Table S3 — ACE2 peptides identified by LC-MS/MS in the 60 kDa protein band. (DOC) [file pone.0085958.s006.doc]

# Supporting Information

**Table S3. ACE2 peptides identified by LC-MS/MS in the 60 kDa protein band**

| **Position** | **Sequence** | **Ions score*^a^*** | **Observed*^b^*** | **Mr(expt)*^c^*** | **Mr(calc)*^d^*** | **ppm*^e^*** | **Miss*^f^*** |
| --- | --- | --- | --- | --- | --- | --- | --- |
| 18 – 26 | QSLTEENAK | 16 | 501.74 | 1001.47 | 1001.47 | 3 | 0 |
| 69 – 78 | WSAFYEEQSK | 33 | 637.79 | 1273.56 | 1273.56 | -2 | 0 |
| 79 – 94 | **TAQSFSLQEIQTPIIK** | 71 | 902.50 | 1802.98 | 1802.98 | 0 | 0 |
| 79 – 95 | TAQSFSLQEIQTPIIKR | 34 | 654.04 | 1959.09 | 1959.08 | 6 | 1 |
| 96 – 112 | **QLQALQQSGSSALSADK** | 101 | 866.45 | 1730.88 | 1730.88 | 0 | 0 |
| 96 – 114 | QLQALQQSGSSALSADKNK | 34 | 658.68 | 1973.03 | 1973.02 | 6 | 1 |
| 115 - 131 | **QLNTILNTMSTIYSTGK** | 49 | 942.99 | 1883.97 | 1883.97 | 0 | 0 |
| 175 - 187 | QLRPLYEEYVVLK | 26 | 816.95 | 1631.89 | 1631.89 | -1 | 0 |
| 220 - 228 | **NQLIEDVER** | 53 | 558.29 | 1114.57 | 1114.56 | 4 | 0 |
| 307 - 313 | IFQEAEK | 17 | 432.73 | 863.44 | 863.44 | 3 | 0 |
| 394 - 416 | NGANEGFHEAVGEIMSLSAATPK | 35 | 782.71 | 2345.11 | 2345.10 | 6 | 0 |
| 420 - 441 | **SIGLLPSDFQEDSETEINFLLK** | 62 | 1248.14 | 2494.26 | 2494.25 | 7 | 0 |
| 442 - 458 | **QALTIVGTLPFTYMLEK** | 42 | 971.02 | 1940.03 | 1940.03 | 0 | 0 |
| 519 - 534 | **TIYQFQFQEALCQAAK** | 74 | 973.48 | 1944.95 | 1944.94 | 6 | 0 |
| 557 - 568 | **MLSLGNSEPWTK** | 46 | 689.84 | 1377.66 | 1377.66 | -1 | 0 |
| 569 - 577 | **ALENVVGAR** | 59 | 464.77 | 927.52 | 927.51 | 5 | 0 |

**Note**: ACE2 peptide sequences, positions, and matched scores were determined with LC-MS/MS analysis, and statistically significant matched peptide sequences were underlined and shown in bold. The cutoff ions score (p<0.05) was 35, in which individual peptide matches with score above 35 are considered statistically significant. False discovery rate for this search was 3.44%. MS error tolerance used was ±7 ppm.

*^a^* a measure of statistical significance of how well the observed MS/MS spectrum matches to the theoretical spectrum of the stated peptide.

*^b^* experimentally determined mass to charge ratio of the ionized molecule (m/z).

*^c^* experimentally determined mass.

*^d^* theoretical calculated mass of the matched peptide.

*^e^* "parts per million" difference (error) between the experimentally observed mass and the theoretically calculated mass.

*^f^* number of missed cleavage sites.
